# Supplementary material for: The oldest record of Saurosphargiformes (Diapsida) from South China could fill an ecological gap in the Early Triassic biotic recovery
Source: PeerJ. 2022 Jul 14;10:e13569. doi: 10.7717/peerj.13569 (PMC9288826; doi:10.7717/peerj.13569)
Supplement: Supplemental Information 4 [file peerj-10-13569-s004.pdf]

The oldest record of Saurosphargiformes (Diapsida) from South China could fill and ecological gap in the Early Triassic biotic recovery

Cheng Long; Benjamin C. Moon; Yan Chunbo; Ryosuke Motani; Jiang Da-yong; An Zhihui; Fang Zicheng

Description of Supplementary Materials 1
Estimation of body length 2
Supplemental Phylogenetic Methods 2
  Modifications to Matrix of Li et al. (2014) 3
  Analyses in TNT 4
  MrBayes Analyses 5
  Character lists 7
    Analysis 1: modified matrix and characters from Li et al. (2014) 7
    Analyses 2 and 3: modified matrix from Scheyer et al. (2017) with characters from Chen et al. (2014) 12
Reconstructed apomorphies 20
  Analysis 1 20
  Analysis 2 27
  Analysis 3 34
References 42
Supplemental Figures 43

Description of Supplementary Materials

This supplemental document details the methods and additional results to support the main text. The PDF is compiled from the markdown plain text files using pandoc and ConTeXt. The easiest way is with the makefile, typing in the command line:

```
make supplemental_mateiral.pdf
```

`supplemental_material.pdf` The typeset supplemental materials document compiled from `supplemental_material.md` with the accompanying Makefile (using pandoc and ConTeXt).

> Additional materials are contained in the associated ZIP archive: `Cheng_etal_Pomolispondylus_supplement.zip`.

`supplemental_material.md` This markdown document.

`Makefile` Make file for compiling the supplemental material PDF and cleaning the main folder.

`supplemental_references.bib` BibLaTeX file of references for the supplemental materials.

`peerj.csl` Citations Style Language XML file for compiling citations within the supplemental materials with pandoc.

`Analysis*.nex` NEXUS files produced in Mesquite for each of three analyses described below.

`/figures/` Folder of supplemental figures included in the supplemental material document.

`/tnt_analyses/` Scripts, input files, and output files from the maximum parsimony analyses in TNT. Methods and scripts are described below (Analyses in TNT). Files include:

- `*.tnt`: TNT formatted matrix file.
- `*.run`: script files for the analyses.

- `*_log.out`: plain text logfile output from the whole script and from each analysis with reconstructed apomorphies.
- `*_trees.nex`: all most parsimonious trees resulting from the tree search.
- `*_bremer.nex` and `*_bootstrap.nex`: strict consensus trees with support values.

`/mrbayes_analyses/` Scripts, input files, and output files from the bayesian inference analyses in MrBayes. methods and scripts are described below (MrBayes Analyses). These results are separated into the three additional ZIP files: `Cheng_etal_analysis[1,2,3]_mrbayes.zip`. Files include:

- `Analysis*.nex`: MrBayes formatted NEXUS files used to build the analysis runfile.
- `nexus-*.nex`: NEXUS file used to run the MrBayes analysis with either gamma or log-normal model.
- `submission_script.sh`: Bash script used to submit to BluePebble HPC.
- `mb-*.sh`: Bash scripts to start the BluePebble HPC analysis for the two models.
- `*-gamma-infinity-out` and `*-lnorm-infinity-out`: plain text log files output from each analysis with the convergence statistics and stepping-stone analysis results.
- `*.con.tre`: majority-rule consensus tree with support values resulting from each analysis.

`/mrbayes_analyses/scripts/` Python script used to build multiple runfiles for MrBayes analyses with model testing. Call this using the following (for example):

```
cd ./Analysis1-Li_etal_2014_Geol_Magaz-modified/
python ../scripts/analysis_mrBayesScript.py
Analysis1-Li_etal_2014_Geol_Magaz-modified.nex
../scripts/analysis_mrBayesBlock.txt
../scripts/analysis_mrBayesDistributions.txt
../scripts/analysis_mrBayesPriors.txt
```

This second command calls the script (`analysis_mrBayesScript.py`) with four arguments:

1. NEXUS file name: formatted for MrBayes but without a MrBayes block.
2. `analysis_mrbayesBlock.txt`: plain text file containing the MrBayes block. The flags `$distribution` and `$prior` will be replaced.
3. `analysis_mrBayesDistributions.txt`: plain text file with a line-separated list of distribution models implemented in MrBayes.
4. `analysis_mrBayesPriors.txt`: plain text files with a line-separated list of prior distrbution values (in this case the values for the symmetrical Dirichlet hyperprior).

Estimation of body length

Body length is estimated by comparing humeral lengths among several Early and Middle Triassic diapsid taxa (supplemental tbl. S1).

Supplemental Phylogenetic Methods

We assessed the relationships of *Pomolispondylus biani* by adding it to three phylogenetic analyses using the following data matrices:

Table S1 Estimates of the presacral length (or equivalent) of *Pomolispondylus biani* based on comparisons of humeral length with other Saurosphargiformes and *Eusaurosphargis*.

Taxon	Specimen	Length (mm)	Scaling factor	Total presacral length (mm)	Estimated presacral length (mm) of *Pomolispondylus biani*
Pomolispondylus biani		31.5			
Largocephalosaurus qianensis	IVPP V 15638	164	5.21	800	154
	GMPKU-P-1532-B (B)	184	5.84	1175	201
Sinosaurosphargis yuguiensis	ZMNH M 8797	100	3.17	470 (carapace length)	148
Eusaurosphargis dalsassoi	PIMUZ A/III 4380	15.9	0.505	106	210
Dianmeisaurus gracilis	IVPP V 18630	18.0	0.571	188	329
Diandongosaurus acutidentatus	NMNS00093-F034398	20.0	0.635	170	268
				**Mean length estimate (mm)**	**218**

1. A modified matrix from Li et al. (*2014*) with 42 operational taxonomic units (OTUs) coded for 159 characters (see below for modifications). *Pomolispondylus biani* was coded for 41/159 characters (25.8%) (supplemental figs S1, S4, S6).
2. The matrix of Scheyer et al. (*2017*) with 43 OTUs coded for 213 characters. *Pomolispondylus biani* was coded for 29/213 characters (13.6%) (supplemental figs S2, S8, S10).
3. The matrix of Scheyer et al. (*2017*) with 43 OTUs coded for 213 characters with marine-related characters changed to uncertainty (following Chen et al. (*2014*)). *Pomolispondylus biani* was coded for 23/213 characters (10.8%) (supplemental figs S3, S12, S14).

We analysed these matrices in a parsimony framework using TNT version 1.5 (February 2022) for Unix (*Goloboff & Catalano, 2016*) and with Bayesian inference using MrBayes version 3.2.7a (*Ronquist et al., 2012*). The matrix was assembled using Mesquite version 3.70(940) (*Maddison & Maddison, 2021*).

*Modifications to Matrix of Li et al. (*2014*)*

The matrix of Li et al. (*2014*) was modified in two characters:

- Character 102: state 2 corrected from previously state 3.
  - Thyroid fenestra (0) absent; (1) present; (2) reduced.
- Character 141: new state 1 now indicates presence of small crest; original characters 2 and 3 combined into new character 3.
  - Proximal curve of the dorsal rib is (0) smooth; (1) with slight crest developed; (2) with developed articular surface for the centrum; (3) with distinct, fan-shaped uncinate process at the flange or spinous process on the concave edge.

The matrix was also coded for three additional OTUs: *Pomolispondylus biani* gen. et sp. nov., *Hanosaurus*, and Hupehsuchia.

Analyses in TNT

We used a new technology search invoking tree fusing, sectorial searching, and the parsimony ratchet (supplemental figs S1–S3) (*Goloboff, 1999*; *Nixon, 1999*).

```
mxram 1024;
proc Analysis1-Li_etal_2014_Geol_Magaz-modified.tnt;
report +/1; taxname =; naked -;
hold 10000;
xmult: hits 40 fuse 5 rat 200 css xss;
```

Each analysis has the following steps:

1. Load relevant data
2. New technology search:
   a. hit shortest length 40 times
   b. 5 sets of tree fusing
   c. 200 rounds of parsimony ratchet
   d. consensus- and exclusive-based sectorial searching
   e. Tree bisection-reconnection on found trees
3. Compute consensus
4. Bremer values calculated using TBR searching up to 10 extra steps
5. Bootstrap resampling analysis:
   a. 100 replicates
   b. Each bootstrap replicate with 100 replicates of classic heuristic search
6. Print and list common synapomorphies.

```
proc %1;
xmult; bbreak;
export - %1_trees.nex;
run STATS.run;
nelsen *; tchoose {strict};
sub 10; proc %1_trees.nex;
ttags =; bsupport !!+0 1.;
export - %1_bremer.nex;
sub 0; tchoose {strict};
ttags-;ttags=;
resample boot replic 100 frequency nogc from 0 [mu100=ho1;];
export - %1_bootstrap.nex;
ttags -; keep 0;
log/;
log %1_log.out;
proc %1_trees.nex;
apo [0.; apo [-0.;
keep0;
log/;
log +tnt_analyses_log.out;
```

A single script to complete all analyses in included in `tnt_analyses/tnt_analyses.run`, with the analysis commands themselves called from `tnt_analyses/analysis.run`.

Summary statistics for each analysis are shown in supplemental tbl. S2. Resulting strict consensus trees are shown in supplemental figs S1–S3.

Table S2 Summary results of three phylogenetic analyses completed in TNT version 1.5. Number of most parsimonious trees (**MPTs**); length in steps; **CI**, consistency index; **RI**, retention index.

Analysis	Original publication	No. MPTs	Length	CI	RI
1	Li et al. (*2014*)	2	608	0.362	0.658
2	Scheyer et al. (*2017*)	32	851	0.306	0.604
3	Scheyer et al. (*2017*), uncertain marine characters	6	814	0.314	0.580

MrBayes Analyses

We also analysed the same three matrices in a Bayesian inference framework implemented in MrBayes version 3.2.7a and run on the BluePebble cluster at the University of Bristol (Advanced Computing Research Centre). For each analysis we tested between two models of rate evolution (with gamma- and log-normal-distributions) using marginal likelihoods calculated by stepping-stone analysis (*Xie et al., 2011*) following the primary tree search (supplemental figs S4, S6, S8, S10). A symmetrical Dirichlet distribution fixed at infinity was used as the prior for all analyses. For each run, commands were added in a `mrbayes` block by a custom Python script, and analyses were run on the University of Bristol BluePebble cluster. Convergence was assessed by reaching both estimated sample size > 200 and potential scale reduction factor approaching 1.0 (supplemental tbl. S3), as well as visually checking the trace plots with the R version 4.1.0 (*R Core Team, 2021*) and the package CODA version 0.19.4 (supplemental figs S5, S7, S9, S11, S13, S15) (*Plummer et al., 2006*).

Each analysis had the following steps:

1. Set likelihood model and parameter linking.
2. Set prior distribution.
3. MCMC analysis with settings:
   a. 5×10^7 generations
   b. stop when PSRF < 0.007
   c. heating temperature 0.1
   d. 25% relative burn-in
   e. 2 runs each with 8 chains
   f. 4 swaps each swapping generation
   g. sample and print every 1000 generations.
4. Summarize parameters and trees.
5. Stepping stone analysis with settings:
   a. 3.1×10^6 generations
   b. 30 steps
   c. $\alpha = 0.4$.

```
set autoclose=yes nowarn=yes;
outgroup Ancestor;
delete Hanosaurus Ichthyopterygia;
prset applyto=(all) symdirihyperpr=fixed(infinity);
lset rates=gamma datatype=standard coding=variable;
mcmcp temp=0.1 ngen=50000000 mcmcdiagn=yes stoprule=yes stopval=0.007
relburnin=yes burninfrac=0.25 printfreq=1000 samplefreq=1000 nswaps=4
nruns=2 nchains=8 savebrlens=yes;
mcmc filename=gamma-infinity;
sump filename=gamma-infinity;
sumt filename=gamma-infinity;
ss ngen=3100000 nsteps=30 stoprule=no alpha=0.4 filename=gamma-infinity-ss;
```

```
quit;
```

Each of the NEXUS files are included in subfolders within `mrbayes_analyses`, with a bash submission script (for SLURM submission system) in `mrbayes_analyses/submission_script.sh`, which calls subordinate submission scripts in each analysis subfolder.

The following example R code was used to plot each analysis trace.

```
library(coda)
files <- c("lnorm-infinity.run1.p", "lnorm-infinity.run2.p")
mcmc_data <- lapply(
  files,
  read.table,
  sep = "\t",
  header = TRUE,
  skip = 1,
  row.names=1
)
mcmc_data <- lapply(mcmc_data, as.mcmc)
mcmc_data <- as.mcmc.list(mcmc_data)

cairo_pdf(
  "analysis1-lnorm-trace.pdf",
  width = 7,
  height = 10
)
plot(mcmc_data)
dev.off()
```

Summary convergence statistics for each run in MrBayes are shown in supplemental tbl. S3.

Table S3 Summary convergence statistics for analyses 1–3 in MrBayes each with a gamma- and log-normal distribution model of rate evolution. Convergence is indicated by the average standard deviation of split frequencies (ASDF) approaching 0.0 (the run is stopped at ASDF < 0.007), the estimated sample size (ESS) exceeding 200, and average potential scale reduction factor (PSRF) approaching 1.0. ASDF is a measure of the recovery of each split in the tree topology was recovered in the MCMC analysis. ESS and PSRF summarize parameters of the model (tree length for both models; alpha for gamma distribution; sigma for log-normal distribution). NB Average values are shown for ASDF and PSRF while minimum values are shown for ESS.

Gamma	ASDF	PSRF	Tree length ESS	alpha ESS
1	0.006966	1.000	2145.19	2067.76
2	0.006958	1.000	1679.85	2002.38
3	0.006964	1.000	2469.89	2862.00

Log-normal	ASDF	PSRF	Tree length ESS	sigma ESS
1	0.006964	1.000	1207.31	1119.95
2	0.006900	1.000	2145.03	2037.70
3	0.006984	1.000	2873.09	3208.41

The following marginal likelihood values (supplemental tbl. S4) resulted from each stepping stone analysis. Model selection uses the categories defined by Kass & Raftery (*1995*). In each case there is not enough evidence to prefer one model over the other, although gamma-distributed rates of evolution are generally preferred.

Table S4 Marginal likelihoods and Bayes factors tests between two models of state distribution for each of three phylogenetic analyses completed in MrBayes version 3.2.7a. The higher-likelihood model in each analysis is indicated in italics.

Analysis	Gamma distribution	Log-normal distribution	2 × Bayes factor
1	-2571.27	*-2569.95*	2.64
2	-3322.78	*-3319.47*	6.62
3	-3183.15	*-3182.94*	0.42

Resulting topologies from the six MrBayes runs (three analyses each with two rates models) are shown in supplemental figs S4, S6, S8, S10, S12, S14. Trace plots of the main parameters for each of these six runs are included in supplemental figs S5, S7, S9, S11, S13, S15.

Character lists

Character lists for the phylogenetic matrices are included here. Note that in TNT character numbers begin from 0.

*Analysis 1: modified matrix and characters from Li et al. (*2014*).* This incorporates the changes described above (Modifications to Matrix of Li et al. (*2014*))

1. Premaxillae small (0) or large (1), forming most of snout in front of external nares.
2. Premaxilla without (0) or with (1) postnarial process, excluding maxilla from posterior margin of external naris.
3. Snout unconstricted (0), constricted (1), or tapering/pointed (2).
4. Temporal region of skull relatively high (0) or strongly depressed (1).
5. Nasals shorter (0) or longer (1) than frontal(s).
6. Nasals not reduced (0), reduced (1), or absent (2).
7. Nasals do (0) or do not (1) enter external naris. This character becomes an autapomorphy of some *Cymatosaurus* with the nasal being absent in *Augustasaurus*, *Pistosaurus*, and plesiosaurs. As such, the character is uninformative, and hence ignored in the analysis in Li et al. (*2011*). The nasal is present in the pistosaur *Yunguisaurus* and informative as included in this analysis.
8. Nasals paired, meeting in dorsomedial suture (0), fused (1), or separated from one another by nasal processes of the premaxillae extending back to the frontal bone(s) (2). Taxa which have lost the nasals, but in which the premaxilla extends backwards to contact or enter in between the frontal(s) are coded (2).
9. Lacrimal present and enters the external naris (0) or remains excluded from the external naris by a contact of maxilla and nasal (1), or lacrimal absent (2).
10. Prefrontal and postfrontal separated by the frontal along the dorsal margin of the orbit (0), or a contact of prefrontal and postfrontal excludes the frontal from the dorsal margin of the orbit (1).
11. Dorsal exposure of prefrontal large (0) or reduced (1).
12. Preorbital and postorbital region of skull: of subequal length (0), preorbital region distinctly longer than postorbital region (1), postorbital region distinctly longer (2).
13. Upper temporal fenestra absent (0), present and subequal in size or slightly larger than the orbit (1), present and distinctly larger than orbit (2), present and distinctly smaller than orbit (3); secondarily closed or nearly closed (4).
14. Frontal(s) paired (0) or fused (1) in the adult.
15. Frontal(s) without (0) or with (1) distinct posterolateral processes.
16. Frontal widely separated from the upper temporal fenestra (0), narrowly approaches the upper temporal fenestra (1), or enters the anteromedial margin of the upper temporal fenestra (2).
17. Parietal(s) paired (0), fused in their posterior part only (1), or fully fused (2) in adult.

18. Pineal foramen close to the middle of the skull table (0), weakly displaced posteriorly (1), strongly displaced posteriorly (2), displaced anteriorly (3), or absent (4).
19. Parietal skull table broad (0), weakly constricted (1), strongly constricted (at least posteriorly) (2), or forming a sagittal crest (3).
20. Postparietals present (0) or absent (1).
21. Tabulars present (0) or absent (1).
22. Supratemporals present (0) or absent (1).
23. Jugal extends anteriorly along the ventral margin of the orbit (0), is restricted to a position behind the orbit but enters the latter's posterior margin (1), or is restricted to a position behind the orbit without reaching the latter's posterior margin (2).
24. Posterior extent of the jugal restricted to a level in front of the midpoint of the longitudinal diameter of the upper temporal fenestra (0), or jugal extends backwards nearly to the posterior end of the skull (1).
25. Jugal remains excluded from (0) or enters (1) the upper temporal arch behind the maxilla.
26. Postfrontal large and plate-like (0), with distinct lateral process overlapping the dorsal tip of the postorbital (1), or postfrontal with reduced lateral process and hence more of an elongate shape (2).
27. Lower temporal fenestra absent (0), present and closed ventrally (1), present but open ventrally (2). This is the only multistate character used ordered in Li et al., 2011 but not in this analysis.
28. Squamosal descends to (0) or remains broadly separated from (1) ventral margin of skull.
29. Quadratojugal present (0) or absent (1).
30. Quadratojugal with (0) or without (1) anterior process.
31. Occiput with paroccipital process forming the lower margin of the post-temporal fenestra and extending laterally (0), paroccipital processes trending posterolaterally (1), or occiput plate-like with no distinct paroccipital process and with strongly reduced post-temporal fenestrae (2).
32. Squamosal without (0) or with (1) distinct notch to receive distal tip of paroccipital process.
33. Mandibular articulation approximately at level with occipital condyle (0) or displaced to a level distinctly behind occipital condyle (1), or positioned anterior to the occipital condyle (2).
34. Exoccipitals do (0) or do not (1) meet dorsal to the basioccipital condyle.
35. Supraoccipital exposed more or less vertically on occiput (0), or exposed more or less horizontally at posterior end of parietal skull table (1); supraoccipital U-shaped (2) (*Storrs & Taylor, 1996*; *Storrs, 1997*).
36. Occipital crest absent (0), present but squamosals do not meet behind parietal (1), present and squamosals meet behind parietal (2).
37. Quadrate with straight posterior margin (0) or quadrate shaft deeply excavated (concave) posteriorly (1).
38. Quadrate covered by squamosal and quadratojugal in lateral view (0), or quadrate exposed in lateral view (1), squamosal suspension box-like (2) (*Storrs & Taylor, 1996*; *Storrs, 1997*).
39. Dorsal wing of epipterygoid approximately as broad as its base (0) or narrower than its base (1).
40. Lateral conch on quadrate absent (0) or present (1).
41. Palate kinetic (0) or akinetic (1).
42. Basioccipital tubera free (0) or in complex relation to the pterygoid, as they extend ventrally (1) or laterally (2).
43. Suborbital fenestra absent (0) or present (1). *Paraplacodus* is coded fide Zanon; thalatttosaurs are coded on *Askeptosaurus* (*fide Kuhn-Schnyder, 1971*).
44. Pterygoid flanges well developed and transversely oriented (0), well developed and longitudinally oriented (1) or strongly reduced (2).

45. Premaxilla enters margin of internal naris (0) or is excluded (1).
46. Ectopterygoid present (0) or absent (1).
47. Internal carotid passage enters basicranium (0) or quadrate ramus of pterygoid (1).
48. Retroarticular process of lower jaw absent (0) or present (1).
49. Distinct coronoid process of lower jaw absent (0) or present (1).
50. Surangular without (0) or with (1) strongly projecting lateral ridge defining the insertion area for superficial adductor muscle fibers on the lateral surface of the lower jaw.
51. Mandibular symphysis short (0), somewhat enforced (1), or elongated and 'scoop'-like (2).
52. Splenial bone enters the mandibular symphysis (0), or remains excluded therefrom (1).
53. Teeth set in shallow or deep sockets (0) or superficially attached to bone (1).
54. Anterior (premaxilla and dentary) teeth upright (0) or strongly procumbent (1).
55. Premaxilla and anterior dentary fangs absent (0) or present (1).
56. One or two caniniform teeth present (0) or absent (1) on maxilla.
57. Maxillary tooth row restricted to a level in front of the posterior margin of the orbit (0), or extends backwards to a level below the posterior corner of the orbit and/or the anterior corner of the upper temporal fenestra (1), or extends backwards to a level below the anterior one third to one half of the upper temporal fenestra (2).
58. Teeth on dermal palate (pterygoid flange) present (0) or absent (1).
59. Vertebrae notochordal (0) or non-notochordal (1).
60. Vertebrae distinctly amphicoelous (0), weakly amphicoelous or platycoelous (1) or other (2).
61. Dorsal intercentra present (0) or absent (1).
62. Cervical intercentra present (0) or absent (1).
63. Cervical centra rounded (0) or keeled (1) ventrally.
64. Zygosphene-zygantrum articulation absent (0) or present (1).
65. Sutural facets receiving the pedicels of the neural arch on the dorsal surface of the centrum in the dorsal region are narrow (0) or expanded into a cruciform or 'butterfly-shaped' platform (1).
66. Transverse processes of neural arches of the dorsal region relatively short (0) or distinctly elongated and narrow, narrower than the space between the transverse processes (1) or distinct elongate and broad, much broader than the space between the transverse processes (2) or dorsoventrally broad and extremely elongated, extending laterally and approaching the margin of the trunk (3).
67. Vertebral centrum distinctly constricted in ventral view (0) or with parallel lateral edges (1). The thalattosaurs coding is based on *Nectosaurus*.
68. Distal end of transverse processes of dorsal vertebrae not increasing in diameter (0) or distinctly thickened (1).
69. Zygapophyseal pachyostosis absent (0) or present (1).
70. Pre- and postzygapophyses do not (0) or do (1) show an anteroposterior trend of increasing inclination within the dorsal and sacral region.
71. Cervical ribs without (0) or with (1) a distinct free anterior process.
72. Pachyostosis of dorsal ribs absent (0) or present (1).
73. The number of sacral ribs is two (0); three (1); four or more (2).
74. Sacral ribs with (0) or without (1) distinct expansion of distal head. The thalattosaurs coding is based on *Askeptosaurus*.
75. Sacral (and caudal) ribs or transverse processes sutured (0) or fused (1) to their respective centrum.
76. Cleithrum present (0) or absent (1).
77. Clavicles broad (0) or narrow (1) medially.
78. Clavicles and interclavicle in simple overlapping contact (0), or anteromedioventral end of clavicle embraces lateral tip of interclavicle in a complex contact (1).
79. Clavicles do not meet in front of the interclavicle (0) or meet in an interdigitating antero-

medial suture (1).
80. Clavicles without (0) or with (1) anterolaterally expanded corners.
81. Clavicle applied to the anterior (lateral) (0) or to the medial (1) surface of scapula.
82. Interclavicle rhomboidal (0) or T-shaped (1).
83. Posterior process on (T-shaped) interclavicle elongate (0), short (1), or rudimentary or absent (2).
84. Scapula represented by a broad blade of bone (0), with a constriction separating a ventral glenoidal portion from a posteriorly directed dorsal wing (1), or a rod-shaped structure (2).
85. Dorsal wing or process of the eosauropterygian scapula tapering to a blunt tip (0) or ventrally expanded at its posterior end (1).
86. Supraglenoid buttress present (0) or absent (1).
87. One (0) or two (1) coracoid ossifications.
88. Coracoid of rounded contours with a foramen entirely in the bone (0), slightly waisted (1), strongly waisted (2), with expanded medial symphysis and ridgelike thickening of the bone extending from glenoid facet posteriorly along lateral edge of the bone, coracoid foramen not enlarged (3), with expanded medial symphysis and ridgelike thickening of the bone extending from glenoid facet transversely through the bone, coracoid foramen much enlarged (4), rounded or nearly rounded contours with a foramen laterally open (5).
89. Coracoid foramen enclosed by coracoid ossification (0), or between coracoid and scapula (1). Thalattosaurs are coded on the basis of *Thalattosaurus* (*Nicholls, 1999, fig. 10B*).
90. Pectoral fenestration absent (0) or present (1).
91. Limbs short and stout (0) or long and slender (1).
92. Humerus rather straight (0) or ‘curved’ (1).
93. Deltopectoral crest well developed (0) or reduced (1), or absent (2).
94. Insertional crest for latissimus dorsi muscle prominent (0) or reduced / absent (1).
95. Humerus with prominent (0) or reduced (1) epicondyles.
96. Ectepicondylar groove open and notched anteriorly (0), open without anterior notch (1), closed (2) (i.e., ectepicondylar foramen present), or absent (3).
97. Entepicondylar foramen present (0) or absent (1).
98. Radius shorter than ulna (0), or longer than ulna (1) or approximately of the same length (2).
99. Iliac blade well developed (0), reduced but projecting beyond level of posterior margin of acetabular portion of ilium (1), reduced and no longer projecting beyond posterior margin of acetabular portion of ilium (2), or absent, i.e. reduced to simple dorsal stub (3), or elongate shaft (4) (*Sato et al., 2010*).
100. Pubis with convex (0) or with concave (1) ventral (medial) margin, or with extensive ventromedial symphysis (3).
101. Obturator foramen closed (0) or open (1) in adult. The thalattosaur coding is based on *Askeptosaurus*, or absent (2) (*Sato et al., 2010*).
102. Thyroid fenestra absent (0), present (1), reduced (2).
103. Acetabulum oval (0) or circular (1).
104. Femoral shaft stout and straight (0) or slender and sigmoidally curved (1).
105. Internal trochanter well developed (0) or reduced (1).
106. Intertrochanteric fossa deep (0), distinct but reduced (1), or rudimentary or absent (2).
107. Distal femoral condyles prominent (0) or not projecting markedly beyond shaft (1).
108. Anterior femoral condyle relative to posterior condyle larger and extending further distally (0) or smaller/equisized and of subequal extent distally (1).
109. Perforating artery passes between astragalus and calcaneum (0), or between the distal heads of tibia and fibula proximal to the astragalus (1).
110. Astragalus without (0) or with (1) a proximal concavity.
111. Calcaneal tuber absent (0) or present (1).
112. Foot short and broad (0) or long and slender (1).

113. Distal tarsal 1 present (0) or absent (1).
114. Distal tarsal 5 present (0) or absent (1).
115. Total number of tarsal ossifications four or more (0), three (1) or two (2).
116. Metatarsal 5 long and slender (0) or distinctly shorter than the other metatarsals and with a broad base (1).
117. Metatarsal 5 straight (0) or 'hooked' (1).
118. Mineralized sternum absent (0) or present (1).
119. The medial gastral rib element always only has a single (0) lateral process, or may have a two-pronged lateral process on one side (1), or contributing to the formation of the plastron (2) (for Testudines and *Odontochelys*, after *Wu et al., 2011*).
120. Distinct groove on the posterior aspect of the proximal shoulder region of the dorsal ribs absent (0) or present (1). This character is shared by *Corosaurus* and some Chinese taxa. Since the latter are not included in this analysis, this character is uninformative and hence ignored.
121. Prefrontal without (0) or with (1) slender anteromedial process entering between maxilla and premaxilla.
122. Anteromedial corner of the upper temporal fenestra is not (0), is partially (1), or is fully (2) floored by a descensus from the postorbital, which together with neighbouring elements (postfrontal, parietal) separates it from orbit (*Edinger, 1935*).
123. Squamosal does not (0), or does (1) form a box-like suspensorium (*Storrs, 1997*).
124. Braincase located at posterior end (0), or deeply recessed below (1) parietal skull roof (or parietal sagittal crest).
125. Posterior palatine vacuities (Andrews, 1896; distinct medial emargination [concavity] on quadrate ramus of pterygoid behind palatobasal articulation) absent (0), or present (1).
126. Distal end of ulna not (0), or distinctly (1) expanded (Sander et al., 1997; the short ulna of plesiosaurs with a convex postaxial margin is here treated as not comparable [?]).
127. Subcentral foramina absent (0), present (1).
128. Durophagous dentition, including much enlarged palatine tooth plates absent (0) or present (1).
129. Four or more (0), or three or less (1) premaxillary teeth.
130. Pterygoids longer (0), or shorter (1), than palatines.
131. Median gastral element angulated (0), or straight (1).
132. Less (0), or more (1) than 30 cervical vertebrae, or more than 40 (2).
133. Parapophysis does not (0), or does (1) shift backwards on centrum along the cervical vertebral column.
134. Distal articular surface on transverse processes of dorsal vertebrae oblong (0), or evenly rounded (1).
135. Total number of carpal ossifications more than 3 (0), three (1), or two (2).
136. Osteoderms absent (0), present (1); present, dense and in sutural contact, providing closed dorsal body cover (2).
137. Snout relatively short, rounded (0), or elaborated into distinct rostrum (1).
138. Ascending process of maxilla distinct (0), or reduced (low) (1).
139. Occiput (posterior margin of skull table) nearly straight (0), or deeply concave (excavated) (1).
140. Neural canal evenly proportioned (0), distinctly higher than wide (1) or wider than high (2) in *Saurosphargis* and *Sinosaurosphargis*.
141. Proximal curve of the dorsal rib is smooth (0); with slight crest developed (1); with developed articular surface for the centrum (2); with distinct, dan-shaped uncinate process at the flange or spinous process on the concave edge (3).
142. Ischium a broad, 'kidney-shaped' plate (0), or with concave posterior and anterior margins (1).

143. Lateral gastral rib element straight laterally (0), bent upwards laterally (1). This character is coded [0] for *Helveticosaurus*.
144. body contours (trunk) elongated (0), broad and rounded (1)
145. Dorsal ribs slender (0), dorsal ribs transversely broadened (1), dorsal ribs transversely broadened and in antero-posterior contact with each other, forming closed "rib-basket" (2).
146. Vomer does (0), or does not (1) contact pterygoid.
147. Interpterygoid fenestra present (0), or absent (1).
148. Pterygoids in contact anteriorly (0), separated anteriorly (1).
149. Marginal teeth with convex (0) or concave (1) lingual surface of crown.
150. Snout not truncated (0), truncated, with high anterior margin (1), truncated with external nares confluent (2).
151. Rib articulation located intersegmentally (0), intrasegmentally (1).
152. Carapace absent (0), present (1).
153. Plastron absent (0), present (1).
154. Rhamphotheca absent (0), present (1).
155. Acromial process on scapula absent (0), present (1).
156. Hypoischium absent (0), present (1).
157. Gular projections on epiplastron absent (0), present (1).
158. Lateral most elements of gastral sets widely spaced (0), or closely associated with each other (1) or joining in the formation of plastron (2).
159. Premaxilla does (0), or does not (1) enter the external naris.

Analyses 2 and 3: modified matrix from Scheyer et al. (2017) with characters from Chen et al. (2014).

1. Premaxillae are small [0] or large [1], forming most of snout in front of external nares (*Rieppel, Mazin & Tchernov, 1999*).
2. Premaxilla is without [0] or with [1] postnarial process, restricting the contact of the maxilla to the external nares or even excluding maxilla (*Rieppel, Mazin & Tchernov, 1999*, but character definition modified in the interest of informativeness).
3. Snout is unconstricted [0] or constricted [1] (*Rieppel, Mazin & Tchernov, 1999*).
4. Nasals are shorter [0] or longer [1] than frontals (*Rieppel, Mazin & Tchernov, 1999*).
5. Nasals meet in dorsomedial suture [0] or are separated from one another by nasal processes of the premaxillae extending back to the frontal bone(s) [1] (*Rieppel, Mazin & Tchernov, 1999*).
6. The lacrimal is present and enters the external naris [0], or it is present but remains excluded from the external naris by a contact of maxilla and nasal [1], or it is absent [2] (*Rieppel, Mazin & Tchernov, 1999*).
7. The prefrontal and postfrontal are separated by the frontal along the dorsal margin of the orbit [0], or a contact of prefrontal and postfrontal excludes the frontal from the dorsal margin of the orbit [1] (*Rieppel, Mazin & Tchernov, 1999*).
8. Preorbital and postorbital regions of skull are of subequal length [0]; preorbital region is distinctly longer than postorbital region [1]; postorbital region is distinctly longer [2] (*Rieppel, Mazin & Tchernov, 1999*).
9. Upper temporal fossae are absent [0], present and subequal in size or slightly larger than the orbit [1], present and distinctly larger than the orbit [2], or present and distinctly smaller than the orbit [3] (*Rieppel, Mazin & Tchernov, 1999*).
10. Frontal(s) are without [0] or with [1] distinct posterolateral processes (*Rieppel, Mazin & Tchernov, 1999*).
11. Parietal(s) are paired [0], fused in their posterior part only [1], or fully fused [2] in the adult (*Rieppel, Mazin & Tchernov, 1999*).
12. Pineal foramen is close to the middle of the skull table [0], displaced anteriorly [1], or absent [2] (*Rieppel, Mazin & Tchernov, 1999*, but character definition modified in the interest of informativeness).

13. Parietal skull table is broad [0], weakly constricted [1], strongly constricted (at least posteriorly) [2], or forms a sagittal crest [3] (*Rieppel, Mazin & Tchernov, 1999*).
14. Postparietals are present [0] or absent [1] (*Rieppel, Mazin & Tchernov, 1999*).
15. Tabulars are present [0] or absent [1] (*Rieppel, Mazin & Tchernov, 1999*).
16. The jugal extends backward no farther than to the middle of the cheek region [0] or nearly to the posterior end of the skull [1] (*Rieppel, Mazin & Tchernov, 1999*).
17. Lower temporal fossa is absent [0], present and closed ventrally [1], or present but open ventrally [2] (*Rieppel, Mazin & Tchernov, 1999*, but modified in Rhynchosauria).
18. Squamosal descends to ventral margin of skull [0], reaches only the approximate mid level of the lower temporal fossa [1], or remains distinctly restricted to the dorsal region of the cheek [2] (*Rieppel, Mazin & Tchernov, 1999*, but character state 1 is new).
19. Quadratojugal is present [0] or absent [1] (*Rieppel, Mazin & Tchernov, 1999*).
20. Quadratojugal has [0] or lacks [1] anterior process (*Rieppel, Mazin & Tchernov, 1999*).
21. Supratemporals are present [0] or absent [1] (*Rieppel, Mazin & Tchernov, 1999*).
22. Mandibular articulations are approximately on a level with occipital condyle [0] or displaced to a level distinctly behind occipital condyle [1], or they are positioned anterior to the occipital condyle [2] (*Rieppel, Mazin & Tchernov, 1999*).
23. Exoccipitals do [0] or do not [1] meet dorsal to the basioccipital condyle (*Rieppel, Mazin & Tchernov, 1999*).
24. Supraoccipital is exposed more or less vertically on occiput [0] or more or less horizontally at posterior end of parietal skull table [1] (*Rieppel, Mazin & Tchernov, 1999*).
25. Occipital crest is absent [0] or present [1] (*Rieppel, Mazin & Tchernov, 1999*).
26. Quadrate has straight posterior margin [0] or the quadrate shaft is deeply excavated (concave) posteriorly [1] (*Rieppel, Mazin & Tchernov, 1999*).
27. Quadrate is covered by squamosal and quadratojugal in lateral view [0] or exposed in lateral view [1] (*Rieppel, Mazin & Tchernov, 1999*).
28. Dorsal wing of epipterygoid is broad [0] or narrow [1] (*Rieppel, Mazin & Tchernov, 1999*).
29. Lateral conch on quadrate is absent [0] or present [1] (*Rieppel, Mazin & Tchernov, 1999*).
30. Palate is kinetic [0] or akinetic [1] (*Rieppel, Mazin & Tchernov, 1999*).
31. Suborbital fenestra is absent [0] or present [1] (*Rieppel, Mazin & Tchernov, 1999*).
32. Pterygoid flanges are well developed [0] or strongly reduced [1] (*Rieppel, Mazin & Tchernov, 1999*).
33. Premaxillae enter internal naris [0] or are excluded [1] (*Rieppel, Mazin & Tchernov, 1999*).
34. Ectopterygoid is present [0] or absent [1] (*Rieppel, Mazin & Tchernov, 1999*).
35. Retroarticular process of lower jaw is absent [0] or present [1] (*Rieppel, Mazin & Tchernov, 1999*).
36. Distinct dorsal process of lower jaw formed by the coronoid only is absent [0] or present [1] [Rieppel, Mazin & Tchernov (*1999*), but character definition modified in the interest of informativeness).
37. Splenial bone enters the mandibular symphysis [0] or remains excluded therefrom [1] (*Rieppel, Mazin & Tchernov, 1999*).
38. Tooth implantation is subthecodont [0], thecodont [1], ankylothecodont [2], or teeth are superficially attached to bone [3] (*Rieppel, Mazin & Tchernov, 1999*).
39. One or two caniniform teeth are present [0] or absent [1] on maxilla (*Rieppel, Mazin & Tchernov, 1999*).
40. Vertebrae are notochordal [0] or nonnotochordal [1] (*Rieppel, Mazin & Tchernov, 1999*).
41. Vertebrae are amphicoelous [0], platycoelous [1], or other [2] (*Rieppel, Mazin & Tchernov, 1999*).
42. Dorsal intercentra are present [0] or absent [1] (*Rieppel, Mazin & Tchernov, 1999*).
43. Cervical intercentra are present [0] or absent [1] (*Rieppel, Mazin & Tchernov, 1999*).
44. Zygosphene-zygantrum articulation is absent [0] or present [1] (*Rieppel, Mazin & Tchernov,*

1999).

45. Sutural facets receiving the pedicels of the neural arch on the dorsal surface of the centrum in the dorsal region are narrow [0] or expanded into a cruciform or butterfly-shaped platform [1] (*Rieppel, Mazin & Tchernov, 1999*).
46. Transverse processes of neural arches of the dorsal region are relatively short [0] or distinctly elongated [1] (*Rieppel, Mazin & Tchernov, 1999*).
47. Pre- and postzygapophyses do not [0] or do [1] show an anteroposterior trend of increasing inclination within the dorsal and sacral region (*Rieppel, Mazin & Tchernov, 1999*).
48. Cervical ribs are without [0] or with [1] a distinct free anterior process (*Rieppel, Mazin & Tchernov, 1999*).
49. The number of sacral ribs is two [0], three [1], or four or more [2] (*Rieppel, Mazin & Tchernov, 1999*).
50. Sacral (and caudal) ribs of transverse processes are sutured [0] or fused [1] to their respective centrum (*Rieppel, Mazin & Tchernov, 1999*).
51. Cleithrum is present [0] or absent [1] (*Rieppel, Mazin & Tchernov, 1999*).
52. Clavicles are broad [0] or narrow [1] medially (*Rieppel, Mazin & Tchernov, 1999*).
53. Clavicles are positioned anteroventrally [0] or dorsally [1] to the interclavicle (*Rieppel, Mazin & Tchernov, 1999*, but modified in many taxa).
54. Clavicle is applied to the anterior (lateral) [0] or to the medial [1] surface of scapula (*Rieppel, Mazin & Tchernov, 1999*).
55. Interclavicle anterior process or triangle: [0] present [1] absent (modified *Rieppel, Mazin & Tchernov, 1999*; by *Chen et al., 2014*).
56. Posterior process on interclavicle is elongate [0], short [1], or rudimentary or absent [2] (*Rieppel, Mazin & Tchernov, 1999*, but character definition modified in the interest of informativeness).
57. Supraglenoid buttress is present [0] or absent [1] (*Rieppel, Mazin & Tchernov, 1999*).
58. Coracoid foramen is enclosed by coracoid ossification [0], or lies between coracoid and scapula [1] (*Rieppel, Mazin & Tchernov, 1999*).
59. Pectoral fenestration is absent [0] or present [1] (*Rieppel, Mazin & Tchernov, 1999*).
60. Humerus is rather straight [0] or 'curved' [1] (*Rieppel, Mazin & Tchernov, 1999*).
61. Deltopectoral crest is well developed [0] or reduced [1] (*Rieppel, Mazin & Tchernov, 1999*).
62. Insertional crest for latissimus dorsi muscle is prominent [0] or reduced [1].
63. Humerus has prominent [0] or reduced [1] epicondyles (*Rieppel, Mazin & Tchernov, 1999*).
64. Ectepicondylar groove: [0] open and notched anteriorly; [1] open without notch [2] closed (i.e., extepicondylar foramen present); [3] absent (modified Rieppel, Mazin & Tchernov (*1999*) by Chen et al. (*2014*)). State 3 was added. This was necessary because the groove is completely absent in ichthyopterygians and hupehsuchians, and probably in *Wumengosaurus*.
65. Entepicondylar foramen is present [0] or absent [1] (*Rieppel, Mazin & Tchernov, 1999*).
66. Radius is shorter than ulna [0], longer than ulna [1], or approximately the same length [2] (*Rieppel, Mazin & Tchernov, 1999*).
67. Iliac blade is well developed [0] or reduced [1] (*Rieppel, Mazin & Tchernov, 1999*).
68. Thyroid fenestra is absent [0] or present [1] (*Rieppel, Mazin & Tchernov, 1999*).
69. Acetabulum is oval [0] or circular [1] (*Rieppel, Mazin & Tchernov, 1999*).
70. Femoral shaft is stout and straight [0] or slender and sigmoidally curved [1] (*Rieppel, Mazin & Tchernov, 1999*).
71. Intertrochanteric fossa is deep [0], distinct but reduced [1], or rudimentary or absent [2] (*Rieppel, Mazin & Tchernov, 1999*).
72. Distal femoral condyles are prominent [0] or do not project markedly beyond shaft [1] (*Rieppel, Mazin & Tchernov, 1999*).
73. Anterior femoral condyle relative to posterior condyle is larger and extends further distally [0] or is smaller/equisized and of subequal extent distally [1] (*Rieppel, Mazin & Tchernov,*

1999).

74. Foramen for the supposed passage of the pes artery is present [0] or absent between astragalus and calcaneum [1] (*Rieppel, Mazin & Tchernov, 1999*).
75. Calcaneal tuber is absent [0] or present [1] (*Rieppel, Mazin & Tchernov, 1999*).
76. Distal tarsal 1 is present [0] or absent [1] (*Rieppel, Mazin & Tchernov, 1999*).
77. Distal tarsal 5 is present [0] or absent [1] (*Rieppel, Mazin & Tchernov, 1999*).
78. Total number of tarsal ossifications is four or more [0], or less than four [1] (*Rieppel, Mazin & Tchernov, 1999*, but character definition modified in the interest of informativeness).
79. Metatarsal 5 is long and slender [0] or distinctly shorter than the other metatarsals and with a broad base [1] (*Rieppel, Mazin & Tchernov, 1999*).
80. Metatarsal 5 is straight [0] or 'hooked' [1] (*Rieppel, Mazin & Tchernov, 1999*).
81. Mineralized sternum is absent [0] or present [1] (*Rieppel, Mazin & Tchernov, 1999*).
82. Upper temporal fenestra. Oval in outline and not elongated caudally [0] or elongated caudally with inner surface of parietal and squamosal facing dorsally [1] (*Dilkes, 1998*).
83. Shape of premaxilla. Horizontal ventral margin [0] or down-turned ventral margin [1] (*Dilkes, 1998*).
84. External nares. Separate [0] or single, medial naris [1] (*Dilkes, 1998*).
85. External nares location. Marginal [0] or close to midline [1] (*Dilkes, 1998*).
86. External nares shape. Rounded [0] or elongate [1] (*Dilkes, 1998*).
87. Septomaxilla is present [0] or absent [1] (*Dilkes, 1998*).
88. Form of suture between premaxilla and maxilla above dentigerous margin. Simple vertical or diagonal contact [0] or notch present in maxilla [1] (*Dilkes, 1998*).
89. Postorbital and parietal contact is absent [0] or present [1] (*Dilkes, 1998*).
90. Postfrontal is excluded from upper temporal fenestra [0] or enters upper temporal fenestra [1] (*Dilkes, 1998*).
91. Subtemporal process of jugal is robust with height >50 % of length [0], slender with height <50 % of length (1), or absent [2]. (*Dilkes, 1998*, but character definition modified in the interest of informativeness).
92. The contact between vomer and maxilla is absent [0] or present [1] (*Dilkes, 1998*).
93. Contact between ectopterygoid and jugal. No contact or restricted area of contact approximately equal or less than contact between ectopterygoid and pterygoid [0] or ectopterygoid expanded caudally [1] (modified from *Dilkes, 1998*).
94. Contact between ectopterygoid and maxilla is present [0] or absent [1] (*Dilkes, 1998*).
95. Shape of ectopterygoid along suture with pterygoid. Transversely broad [0], posteroventrally elongate and does not reach lateral corner of transverse flange [1], or posteroventrally elongate and reaches corner of transverse flange [2] (*Dilkes, 1998*).
96. Orientation of basipterygoid processes is anterolateral [0] or lateral [1] (*Dilkes, 1998*).
97. Parasphenoid teeth are present [0] or absent [1] (*Dilkes, 1998*).
98. Paroccipital process ends freely [0] or reaches suspensorium [1] (*Dilkes, 1998*).
99. Palatine teeth are present [0] or absent [1] (*Dilkes, 1998*).
100. Teeth on palatine ramus of pterygoid are present [0] or absent [1] (*Dilkes, 1998*, but character definition modified in the interest of informativeness).
101. Upturned retroarticular process is absent [0] or present [1] (*Dilkes, 1998*).
102. Slender and tapering cervical ribs at low angle to vertebrae are absent [0] or present [1] (*Dilkes, 1998*).
103. Neural arches of mid-dorsals are shallowly excavated [0] or deeply excavated [1] (*Dilkes, 1998*).
104. Most trunk ribs are dichocephalous [0], holocephalous but not clearly articulating with the neural arch only [1], or holocephalous and only articulating with the neural arch [2] (*Dilkes, 1998*, but the separation between states 1 and 2 is new).
105. Second sacral rib is not bifurcate [0], or bifurcate [1] (*Dilkes, 1998*, but character definition

modified in the interest of informativeness).

106. Proximal caudal neural spine height. Moderately tall with height / length > 1.0 and <2.0 [0], low with height/length <1.0 [1], tall with height/length >2.0 and <3.0 [2], or very tall with height/length >3.0 [3] (*Dilkes, 1998*).
107. Ratio of lengths of caudal transverse processes and centra >1.0 [0] or <1.0 [1] (*Dilkes, 1998*, but character definition modified in the interest of informativeness).
108. Distal width of haemal spine is equivalent to proximal width [0], tapering [1], or wider than proximal width [2] (*Dilkes, 1998*).
109. Gastralia are present [0] or absent [1] (*Dilkes, 1998*).
110. Coracoid process is small [0] or large [1] (*Dilkes, 1998*).
111. Cranial margin of interclavicle lacks [0] or has distinct notch [1] (*Dilkes, 1998*).
112. Caudal stem of interclavicle lacks [0] or has distinct expansion [1] (*Dilkes, 1998*).
113. Dorsal margin of ilium is without [0] or with [1] anterior process (*Dilkes, 1998*).
114. Processus lateralis is present [0] or absent [1] (*Dilkes, 1998*).
115. Supratemporal stout and short [0], long and slender, projecting far anteriorly [1], or same as [1] but also contacting the frontal [2] (modified from Nicholls, 1999).
116. Diastema between maxillary and premaxillary teeth absent [0] or present [1] (*Nicholls, 1999*).
117. Anterior (premaxillary and dentary) teeth are upright [0] or strongly procumbent [1] (*Nicholls, 1999*).
118. The maxillary tooth row is restricted to a level in front of the posterior margin of the orbit [0], or it extends backward [1] (*deBraga & Rieppel, 1997*).
119. Scapula is represented by a broad blade of bone [0], is slender, high, and narrow [1], or with a constriction separating a ventral glenoidal portion from a posteriorly directed dorsal wing [2] (*Rieppel, Mazin & Tchernov, 1999*).
120. Internal trochanter is well-developed [0] or reduced [1] (*Rieppel, Mazin & Tchernov, 1999*).
121. Heavy, conical teeth on vomer are absent [0] or present [1] (modified from Nicholls, 1999).
122. Button-like, durophagous teeth are absent [0] or present [1] (*Nicholls, 1999*).
123. One [0] or two [1] coracoid ossifications are present (*Rieppel, Mazin & Tchernov, 1999*).
124. Dentary symphysis is straight [0] or recurved [1] (*Dilkes, 1998*, but character definition modified in the interest of informativeness).
125. Choana palatal exposure: parallel medial border of maxilla [0]; deflected posteromedially [1]; hidden in palatal view [2] (*deBraga & Rieppel, 1997*).
126. Maxilla ascending process between naris and orbit is absent [0] or present [1] (*deBraga & Rieppel, 1997*).
127. Maxilla extends to posterior orbital margin [0] or not [1] (*deBraga & Rieppel, 1997*).
128. Maxilla orbital exposure is absent [0] or present [1] (*deBraga & Rieppel, 1997*).
129. Lacrimal duct: enclosed by lacrimal only [0]; lateral border formed by maxilla [1] (*deBraga & Rieppel, 1997*).
130. Prefrontal/palatine antorbital contact is narrow forming less than one third the transverse distance between the orbits [0], or forming at least one half the distance [1] (*deBraga & Rieppel, 1997*).
131. Postorbital posterior extent: terminates prior to reaching posterior limit of parietal [0]; extends to at least the posterior limit of the parietal [1] (*deBraga & Rieppel, 1997*).
132. Squamosal occipital flange is absent or poorly developed forming only a thin ridge [0], or it is well developed forming a broadly exposed lappet [1] (*deBraga & Rieppel, 1997*).
133. Stapes morphology: robust with its greatest depth exceeding one third of its total length [0]; slender with the length at least four times the depth [1] (*deBraga & Rieppel, 1997*).
134. Stapedial dorsal process: present as ossified process [0]; absent [1] (*deBraga & Rieppel, 1997*).
135. Basi/ parasphenoid ratio: narrowest transverse width no more than 60 % of the maximum length measured from basipterygoid process to posteriormost limit [0]; narrowest part (waist) exceeds 80 % of the length [1] (*deBraga & Rieppel, 1997*).

136. Ventral braincase tubera: absent [0]; present and restricted to basioccipital [1]; present, very large, and restricted to basisphenoid [2] (*deBraga & Rieppel, 1997*).
137. Basioccipital/basisphenoid relationship: floor of braincase with gap between both elements [0]; elements fused to floor brain cavity [1] (*deBraga & Rieppel, 1997*).
138. Sphenethmoid is present [0] or absent [1] (*deBraga & Rieppel, 1997*).
139. Palatal process of pterygoid: extends anterior to the anterior limit of the palatine [0]; forms oblique suture with palatine but process ends before reaching anterior limit of palatine [1]; forms transverse suture with palatine [2] (*deBraga & Rieppel, 1997*).
140. Orientation of transverse flange of pterygoid: directed predominantly laterally [0] or antero-laterally [1] (*deBraga & Rieppel, 1997*).
141. Cultriform process: long exceeding length of parasphenoid body and reaching forward to the level of the posterior limit of the internal nares [0]; short not reaching the level of the internal nares [1] (*deBraga & Rieppel, 1997*).
142. Pterygoid transverse flange extends ventrally below the level of the maxillary tooth row [0] or not [1] (*deBraga & Rieppel, 1997*).
143. Surangular extends anterior to coronoid eminence [0] or terminates prior to reaching eminence [1] (*deBraga & Rieppel, 1997*).
144. Atlantal ribs are ossified [0] or not [1] (*deBraga & Rieppel, 1997*).
145. Humeral torsion: proximal and distal ends of humerus are set off at 45° angles from one another [0]; angle between opposing ends reduced to not more than 20° [1].
146. Humeral distal articulations: distinct trochlea and capitellum [0]; low double condyle [1] (*deBraga & Rieppel, 1997*).
147. Olecranon is well developed [0] or small or absent [1] (*deBraga & Rieppel, 1997*).
148. Metacarpal IV is longer than metacarpal III [0], or equal or shorter [1] (*deBraga & Rieppel, 1997*).
149. Fibula is bowed away from tibia [0] or straight and not bowed away [1] (*deBraga & Rieppel, 1997*).
150. Astragalus/ distal tarsal IV articulation: articulation poorly defined [0], well defined [1], or absent [2] (*deBraga & Rieppel, 1997*).
151. Number of pedal centralia: both lateral and medial centrali present [0]; medial pedal centrale lost [1]; both centralia lost [2] (*deBraga & Rieppel, 1997*).
152. Premaxilla dentition: [0] 4 or more [1] 3 or less [2] edentulous (modified from deBraga & Rieppel (*1997*) by Chen et al. (*2014*)). State 2 was added.
153. Nasals are paired [0], fused [1], or lost (*deBraga & Rieppel, 1997*).
154. Frontal anterior margins: [0] frontal suture with nasal transverse; [1] oblique, pointing antero-laterally; [2] oblique, pointing antero-medially (modified from deBraga & Rieppel (*1997*) by Chen et al. (*2014*)). State 1 was modified and state 2 was added. These two states cannot be considered homologous although both are "oblique".
155. Frontal proportions: length exceeds width by at least four times [0]; length no greater than twice the width [1] (*deBraga & Rieppel, 1997*).
156. Quadrate anterior process: long, extending forward along its sutural contact with the quadrate process of the pterygoid to nearly reach the level of the transverse flange [0]; short, not extending anteriorly beyond 55% the length of the quadrate process of the pterygoid [1] (*deBraga & Rieppel, 1997*).
157. Parietal shelf for adductor musculature is absent [0] or present as shallow excavations on the lateral parietal margin [1] (*deBraga & Rieppel, 1997*).
158. Paroccipital processes extend laterally forming 90° with parasagittal plane [0], or are deflected posterolaterally at an angle of about 20° from the transverse width of the skull [1], or are deflected dorsolaterally at an angle of nearly 45° [2] (from deBraga & Rieppel (*1997*)).
159. Paroccipital processes are slender [0] or heavy with anteroposterior dimension at least one third greater than dorsoventral dimension [1] (*deBraga & Rieppel, 1997*).

160. Prootic/parietal contact is absent [0] or present [1] (*deBraga & Rieppel, 1997*).
161. Medial wall of inner ear is unossified [0] or ossified [1] (*deBraga & Rieppel, 1997*).
162. Interpterygoid vacuity: anterior end tapers sharply [0]; anterior border crescentic [1]; absent [2] (*deBraga & Rieppel, 1997*).
163. Dentition on transverse flange of pterygoid: present as shagreen of teeth [0]; present but with one large distinct row of teeth along the posterior edge of the transverse flange [1]; edentulous [2] (*deBraga & Rieppel, 1997*).
164. Transverse flange lateral margin: posterolateral margin forms sharp edge with anteromedial margin [0]; posterolateral margin merges smoothly into anteromedial margin forming a smoothly convex lateral outline [1] (*deBraga & Rieppel, 1997*).
165. Meckelian fossa faces mediodorsally [0] or dorsally due to greatly expanded prearticular [1] (*deBraga & Rieppel, 1997*).
166. Surangular lateral shelf is absent [0] or present [1] (*deBraga & Rieppel, 1997*).
167. Angular lateral exposure: exposed along one third the lateral face of the mandible [0]; exposed only as a small sliver along the lateral face [1]; absent from lateral aspect [2] (*deBraga & Rieppel, 1997*).
168. Prearticular extends anterior to coronoid eminence [0] or terminates prior reaching eminence [1] (*deBraga & Rieppel, 1997*).
169. Caudal lateral projections (transverse processes) are absent [0] or present [1] beyond fifth caudal (*deBraga & Rieppel, 1997*).
170. Tibia/astragalus articulation: loose fitting [0]; tightly fitting with well developed articulation [1] (from *deBraga & Rieppel, 1997*, but modified in Choristodera).
171. Astragalus and calcaneum: never fused in adult [0]; fused [1]; hinge present [2] (*deBraga & Rieppel, 1997*).
172. Metatarsal V plantal tubercle is absent [0] or present [1] (*deBraga & Rieppel, 1997*).
173. Metatarsal I/IV ratio: I greater than 50% the length of IV [0]; I less than 50% the length of IV [1]. (Modified in Choristodera) (*deBraga & Rieppel, 1997*).
174. Ratio of length of centra of mid-cervical and mid-dorsal vertebrae: < 1.0 [0]; >1.0 [1]; >1.5 [2] (*Dilkes, 1998*).
175. Pubic tubercle: if present small and and directed anteroventrally [0]; large and strongly turned ventrally [1] (*deBraga & Rieppel, 1997*).
176. Supinator process: large angled away from humeral shaft [0]; large confluent with shaft [1]; small or absent [2] (*deBraga & Rieppel, 1997*).
177. Skull roof is without [0] or with [1] distinct posterior emargination (*Müller, 2004*).
178. Frontal widely separated from the upper temporal fossa [0], or narrowly approaches or enters the anteromedial margin margin of the upper temporal fossa [1] (*Rieppel, Mazin & Tchernov, 1999*, but character definition modified in the interest of informativeness).
179. Prefrontal and maxilla do not meet due to a contact of lacrimal and nasal [0], or prefrontal contacts maxilla anteriorly and thereby separates lacrimal and nasal from one another [1] (*Müller, 2004*).
180. Nares are positioned anteriorly [0] or are situated in the central or posterior area of the antorbital skull portion [1] (*Müller, 2004*).
181. Lacrimal enters the orbital margin [0], or remains excluded therefrom due to an external contact between the posteroventral part of the prefrontal and the posterodorsal margin of the maxilla [1] (*Müller, 2004*).
182. Quadratojugal remains restricted to the ventral margin of the cheek [0], or shows a distinct dorsal extension [1] (*Müller, 2004*).
183. Two or more coronoids are present [0] or absent [1] (*deBraga & Rieppel, 1997*).
184. The postorbital is in contact with the supratemporal [0] or not [1] (modified from *deBraga & Rieppel, 1997*).
185. Postorbital: posterior process contacting squamosal long [0] or short [1] (*Bickelmann, Müller*

& Reisz, 2009).

186. Dorsal vertebral count: [0] 19 or more; [1] 18 or fewer (modified from Bickelmann, Müller & Reisz (*2009*) by Chen et al. (*2014*)). The threshold was determined based on histograms derived from the data in Müller et al. (*2010*), where basal amniotes have at least 19 dorsal vertebrae. This value also matches the difference between 24 and 5, the former being the threshold used for the original coding for presacral count, the latter for the cervical count (character 187). See the main text for further explanation.
187. Neck: short with 5 or less vertebrae [0] or long with more than 5 vertebrae [1] (*Bickelmann, Müller & Reisz, 2009*).
188. Ulnare: wider than long [0], longer than wide [1], or as long as wide [2] (*Bickelmann, Müller & Reisz, 2009*).
189. Pachyostosis of dorsal ribs absent [0] or present [1] (*Li et al., 2014*, character 72).
190. Distal end of ulna not [0], or distinctly [1] expanded (Sander et al. 1997; the short ulna of plesiosaurs with a convex margin is here treated as not comparable [?]) (*Li et al., 2014*, character 126).
191. Median gastral element angulated and pointed anteriorly [0], straight [1], or angulated and pointing posteriorly (Li et al. (*2014*) character 131, modified by Chen et al. (*2014*)).
192. Total number of carpal ossifications more than 3 [0], three [1], or two [2] (*Li et al., 2014*, character 135).
193. Osteoderms absent [0], present [1]; present, dense and in a sutural contact, providing closed dorsal body cover [2] (*Li et al., 2014*, character 136).
194. Neural canal evenly proportioned [0], distinctly higher than wide [1], or wider than high [2] in *Saurosphagis* and *Sinosaurosphargis* (*Li et al., 2014*, character 140).
195. Dorsal ribs without [0], or with distinct, fan-shaped uncinate process on the convex margin [1], or on the concave margin [2], or with a distinct crest on the dorsal surface of the shoulder region [3] (*Li et al., 2014*, character 141).
196. Ischium a broad "kidney-shaped" plate [0], or with concave posterior and anterior margins [1] (*Li et al., 2014*, character 142).
197. Dorsal ribs slender [0], dorsal ribs transversely broadened [1], dorsal ribs transversely broadened and in antero-posterior contact with each other, forming closed "rib-basket" [2] (*Li et al., 2014*, character 145).
198. Pterygoids in contact anteriorly [0], separated anteriorly [1] (*Li et al., 2014*, character 148).
199. Marginal teeth with convex [0] or concave [1] lingual surface of crown (*Li et al., 2014*, character 149).
200. Premaxilla is longer than maxilla: [0] false [1] true (*Chen et al., 2014*).
201. Nasal extends anteriorly much beyond external naris: [0] false [1] true (*Chen et al., 2014*).
202. Frontal, butterflyu-shaped with antero- and postero-lateral processes: [0] false [1] true (*Chen et al., 2014*).
203. Supratemporal occipital "lappet": [0] absent [1] present (*Chen et al., 2014*).
204. Large scleral ring filling the orbit: [0] absent [1] present (*Chen et al., 2014*).
205. Angular maximum exposed height relative to surangular: [0] aubequal or angular higher; [1] angular clearly lower (*Chen et al., 2014*).
206. Body compression: [0] subequal or slightly compressed dorso-ventrally; [1] clearly flattened dorso-ventrally; [2] bilaterally compressed (*Chen et al., 2014*).
207. Lateral gastralia, expanded and flat: [0] false [1] true (*Chen et al., 2014*).
208. Strong anticlination of mid-caudal neural spines: [0] absent [1] present (*Chen et al., 2014*).
209. Humerus anterior flange: [0] absent [1] present (*Chen et al., 2014*).
210. Radius anterior flange: [0] absent [1] present (*Chen et al., 2014*).
211. Radiale larger then other carpals: [0] false [1] true (*Chen et al., 2014*).
212. Forelimb digits: [0] at least some divergent; [1] all convergent (*Chen et al., 2014*).
213. Femur, distal and proximal extremities: [0] of subequal lengths; [1] distal one much wider

(*Chen et al., 2014*).

These 24 character states were replaced with uncertain in marine taxa in analysis 3 following Chen et al. (*2014*):

- Character 1, state 1.
- Character 9, state 3.
- Character 11, state 0.
- Character 50, state 0.
- Character 58, state 1.
- Character 61, state 1.
- Character 62, state 1.
- Character 63, state 1.
- Character 67, state 1.
- Character 68, state 1.
- Character 71, state 3.
- Character 72, state 1.
- Character 74, state 1.
- Character 78, state 1.
- Character 106, states 2 and 3.
- Character 114, state 1.
- Character 120, state 1.
- Character 145, state 1.
- Character 151, state 2.
- Character 171, state 0.
- Character 180, state 1.
- Character 186, state 0.
- Character 192, states 1 and 2.
- Character 200, state 1.

Reconstructed apomorphies

The apomorphies below are common to the most parsimonious trees found for each analysis in TNT.

Analysis 1

```
Synapomorphies common to 2 trees
(Node numbers refer to nodes in
consensus)

   Ancestor :
     All trees:
       No autapomorphies:

   Captorhinidae :
     All trees:
       Char. 45: 0 --> 1

   Chelydra :
     All trees:
        Char. 5: 0 --> 2
        Char. 8: 1 --> 2
        Char. 32: 0 --> 2
        Char. 40: 0 --> 1
        Char. 44: 0 --> 1
        Char. 57: 0 --> 1
        Char. 59: 0 --> 2
        Char. 100: 0 --> 1
        Char. 101: 0 --> 1
        Char. 146: 0 --> 1
        Char. 149: 1 --> 2

   Proganochelys :
     All trees:
```

```
    Char. 135: 0 --> 1

Araeoscelidia :
  All trees:
    No autapomorphies:

Younginiformes :
  All trees:
    Char. 26: 02 --> 1
    Char. 48: 0 --> 1

Kuehneosauridae :
  All trees:
    Char. 65: 0 --> 1
    Char. 69: 0 --> 1
    Char. 83: 0 --> 1
    Char. 147: 0 --> 1

Rhynchocephalia :
  All trees:
    Char. 23: 0 --> 1
    Char. 27: 1 --> 0
    Char. 96: 1 --> 0
    Char. 142: 0 --> 1

Squamata :
  All trees:
    Char. 145: 0 --> 1

Rhynchosauria :
  All trees:
    Char. 16: 0 --> 1
    Char. 70: 1 --> 0
    Char. 108: 0 --> 1

Prolacertiformes :
  All trees:
    Char. 52: 0 --> 1
    Char. 81: 1 --> 0
    Char. 141: 0 --> 1

Trilophosaurus :
  All trees:
    Char. 9: 0 --> 1
    Char. 15: 0 --> 1
    Char. 26: 1 --> 0
    Char. 32: 0 --> 2
    Char. 38: 1 --> 0
    Char. 118: 0 --> 2

Choristodera :
  All trees:
    Char. 18: 0 --> 1
    Char. 32: 0 --> 1
    Char. 35: 0 --> 1
    Char. 57: 1 --> 0
    Char. 64: 0 --> 1
    Char. 72: 0 --> 1
    Char. 106: 0 --> 1
    Char. 118: 0 --> 2
    Char. 137: 0 --> 1
    Char. 138: 0 --> 1

Archosauriformes :
  All trees:
    Char. 65: 0 --> 1

Claudiosaurus :
  All trees:
    Char. 29: 0 --> 1
    Char. 43: 0 --> 2
    Char. 70: 0 --> 1
    Char. 92: 0 --> 1
    Char. 93: 0 --> 1
    Char. 94: 0 --> 1
    Char. 106: 0 --> 1

Anaro_Dactylo :
  All trees:
    Char. 79: 0 --> 1

Serpiano_Neustico :
  All trees:
    Char. 25: 1 --> 2
    Char. 71: 0 --> 1

Simosaurus :
  All trees:
    Char. 14: 1 --> 0
    Char. 44: 1 --> 0
    Char. 50: 2 --> 0
    Char. 92: 1 --> 0
    Char. 96: 0 --> 1
    Char. 136: 1 --> 0

Nothosaurus :
  All trees:
    Char. 17: 1 --> 2

Lariosaurus :
  All trees:
    Char. 72: 1 --> 2
```

```
    Char. 114: 1 --> 0
    Char. 118: 1 --> 0

Corosaurus :
  All trees:
    Char. 7: 2 --> 0
    Char. 11: 2 --> 0
    Char. 12: 2 --> 1
    Char. 48: 0 --> 1
    Char. 50: 2 --> 1
    Char. 59: 1 --> 0
    Char. 74: 0 --> 1
    Char. 119: 0 --> 1

Cymatosaurus :
  All trees:
    Char. 2: 0 --> 1
    Char. 5: 0 --> 1
    Char. 18: 1 --> 23
    Char. 91: 1 --> 0
    Char. 92: 1 --> 0
    Char. 93: 1 --> 0
    Char. 94: 1 --> 0
    Char. 118: 1 --> 0
    Char. 121: 0 --> 1

Germanosaurus :
  All trees:
    Char. 15: 0 --> 1
    Char. 16: 2 --> 1

Pistosaurus :
  All trees:
    Char. 16: 0 --> 1
    Char. 44: 1 --> 0
    Char. 84: 0 --> 1

Placodus :
  All trees:
    Char. 4: 0 --> 1
    Char. 17: 3 --> 0
    Char. 41: 0 --> 1
    Char. 69: 0 --> 1
    Char. 134: 1 --> 2

Cyamodus :
  All trees:
    Char. 1: 0 --> 1
    Char. 11: 0 --> 2
    Char. 32: 0 --> 1
    Char. 38: 1 --> 0
    Char. 45: 0 --> 1
    Char. 65: 1 --> 3
    Char. 143: 0 --> 1

Augustasaurus :
  All trees:
    Char. 38: 1 --> 0
    Char. 56: 1 --> 2
    Char. 131: 0 --> 1

Odontochelys :
  All trees:
    Char. 87: 0 --> 2
    Char. 125: 0 --> 1

Thalattosauria :
  All trees:
    Char. 2: 0 --> 2
    Char. 7: 0 --> 2
    Char. 12: 3 --> 4
    Char. 48: 0 --> 1
    Char. 49: 0 --> 1

Yunguisaurus :
  All trees:
    Char. 2: 0 --> 1
    Char. 5: 2 --> 1
    Char. 15: 0 --> 1
    Char. 37: 1 --> 2
    Char. 48: 0 --> 1
    Char. 49: 1 --> 0
    Char. 56: 1 --> 0
    Char. 59: 1 --> 0
    Char. 62: 1 --> 0
    Char. 66: 0 --> 1
    Char. 84: 0 --> 1
    Char. 100: 1 --> 2
    Char. 111: 0 --> 1
    Char. 119: 0 --> 1

Eusaurosphargis_BES_SC_390 :
  All trees:
    Char. 59: 0 --> 1
    Char. 87: 0 --> 5
    Char. 97: 2 --> 0
    Char. 118: 0 --> 1
    Char. 135: 0 --> 1
    Char. 140: 0 --> 1
    Char. 144: 0 --> 1
    Char. 148: 0 --> 1
```

```
Paraplacodus :
  All trees:
    Char. 36: 1 --> 0
    Char. 49: 0 --> 1
    Char. 87: 0 --> 1
    Char. 98: 1 --> 3
    Char. 140: 0 --> 1

Saurosphargis :
  All trees:
    No autapomorphies:

Sinosaurosphargis :
  All trees:
    Char. 68: 0 --> 1
    Char. 135: 1 --> 2
  Some trees:
    Char. 12: 3 --> 4
    Char. 25: 1 --> 0
    Char. 47: 1 --> 0
    Char. 79: 0 --> 1
    Char. 143: 0 --> 1

Largocephalosaurus_polycarpon :
  All trees:
    Char. 72: 0 --> 1
    Char. 95: 0 --> 2
    Char. 112: 0 --> 1

Largocephalosaurus_qianensis :
  Some trees:
    Char. 140: 2 --> 3

Pomolispondylus_biani :
  All trees:
    Char. 66: 0 --> 1
  Some trees:
    Char. 140: 0 --> 1

Plesiosaurus :
  All trees:
    Char. 50: 2 --> 0
    Char. 63: 1 --> 0
    Char. 87: 3 --> 4
    Char. 94: 1 --> 0
    Char. 136: 1 --> 0
    Char. 137: 1 --> 0

Helveticosaurus :
  All trees:
    Char. 97: 2 --> 1
    Char. 114: 12 --> 3
    Char. 134: 1 --> 2

Hupehsuchia :
  All trees:
    Char. 3: 0 --> 1
    Char. 8: 2 --> 1
    Char. 19: 1 --> 0
    Char. 40: 0 --> 1
    Char. 42: 1 --> 0
    Char. 45: 0 --> 1
    Char. 81: 1 --> 0
    Char. 95: 0 --> 3
    Char. 108: 1 --> 0
    Char. 142: 0 --> 1
  Some trees:
    Char. 87: 0 --> 5

Hanosaurus :
  All trees:
    Char. 4: 0 --> 1
    Char. 13: 0 --> 1
    Char. 14: 1 --> 0
    Char. 71: 0 --> 1
    Char. 88: 1 --> 0
    Char. 103: 1 --> 0
    Char. 141: 1 --> 0
    Char. 148: 0 --> 1

Ichthyopterygia :
  All trees:
    Char. 4: 0 --> 1
    Char. 8: 2 --> 0
    Char. 14: 1 --> 0
    Char. 21: 1 --> 0
    Char. 25: 1 --> 0
    Char. 30: 1 --> 0
    Char. 37: 1 --> 0
    Char. 44: 1 --> 0
    Char. 47: 1 --> 0
    Char. 50: 0 --> 1
    Char. 61: 1 --> 0
    Char. 70: 1 --> 0
    Char. 95: 0 --> 3
    Char. 103: 1 --> 0
    Char. 133: 0 --> 1
    Char. 136: 0 --> 1

Node 43 :
  All trees:
    No synapomorphies
```

```
Node 44 :
  All trees:
    Char. 111: 1 --> 0
    Char. 150: 0 --> 1
    Char. 151: 0 --> 1
    Char. 153: 0 --> 1
    Char. 154: 0 --> 1

Node 45 :
  All trees:
    Char. 11: 1 --> 2
    Char. 12: 1 --> 0
    Char. 66: 0 --> 1
    Char. 76: 1 --> 0
    Char. 83: 0 --> 2
    Char. 88: 0 --> 1
    Char. 105: 1 --> 0
    Char. 106: 0 --> 1
    Char. 117: 1 --> 0
    Char. 143: 0 --> 1
    Char. 149: 0 --> 1
    Char. 152: 0 --> 1
    Char. 157: 0 --> 2

Node 46 :
  All trees:
    Char. 27: 0 --> 1
    Char. 58: 0 --> 1
    Char. 60: 0 --> 1
    Char. 96: 0 --> 1
    Char. 108: 0 --> 1
    Char. 113: 0 --> 1
    Char. 116: 0 --> 1

Node 47 :
  All trees:
    Char. 36: 0 --> 1
    Char. 37: 0 --> 1
    Char. 115: 0 --> 1

Node 48 :
  All trees:
    Char. 8: 0 --> 1
    Char. 55: 0 --> 1
    Char. 75: 0 --> 1
    Char. 76: 0 --> 1
    Char. 85: 0 --> 1
    Char. 102: 0 --> 1
    Char. 103: 0 --> 1
    Char. 105: 0 --> 1
    Char. 107: 0 --> 1

Node 49 :
  All trees:
    Char. 11: 0 --> 1
    Char. 12: 0 --> 1
    Char. 14: 0 --> 1
    Char. 25: 0 --> 1
    Char. 42: 0 --> 1
    Char. 51: 0 --> 1
    Char. 62: 0 --> 1
    Char. 81: 0 --> 1
    Char. 111: 0 --> 1
    Char. 117: 0 --> 1

Node 50 :
  All trees:
    Char. 11: 1 --> 0
    Char. 39: 0 --> 1

Node 51 :
  All trees:
    Char. 29: 0 --> 1
    Char. 38: 0 --> 1
    Char. 57: 0 --> 1

Node 52 :
  All trees:
    Char. 48: 0 --> 1
    Char. 52: 0 --> 1
    Char. 138: 0 --> 1

Node 53 :
  All trees:
    Char. 4: 1 --> 0
    Char. 18: 0 --> 3

Node 54 :
  All trees:
    Char. 23: 0 --> 1
    Char. 97: 2 --> 0

Node 55 :
  All trees:
    Char. 26: 2 --> 1
    Char. 29: 1 --> 0

Node 56 :
  All trees:
    Char. 1: 0 --> 1
    Char. 4: 0 --> 1
    Char. 108: 1 --> 0
```

```
Node 57 :
  All trees:
    Char. 0: 0 --> 1
    Char. 30: 0 --> 1
    Char. 70: 0 --> 1

Node 58 :
  All trees:
    Char. 17: 3 --> 0
    Char. 30: 1 --> 2
    Char. 34: 0 --> 1
    Char. 45: 0 --> 1
    Char. 68: 0 --> 1
    Char. 73: 0 --> 1
    Char. 92: 1 --> 0

Node 59 :
  All trees:
    Char. 63: 0 --> 1
    Char. 64: 0 --> 1
    Char. 77: 0 --> 1
    Char. 80: 0 --> 1
    Char. 89: 0 --> 1
    Char. 91: 0 --> 1

Node 60 :
  All trees:
    Char. 27: 1 --> 0
    Char. 78: 0 --> 1
    Char. 87: 0 --> 2
    Char. 96: 1 --> 0
    Char. 141: 0 --> 1

Node 61 :
  All trees:
    Char. 40: 0 --> 1
    Char. 72: 0 --> 1
    Char. 105: 1 --> 2
    Char. 112: 0 --> 1
    Char. 114: 0 --> 12
    Char. 134: 0 --> 1

Node 62 :
  All trees:
    Char. 8: 1 --> 2
    Char. 12: 1 --> 3
    Char. 43: 0 --> 2
    Char. 92: 0 --> 1
    Char. 93: 0 --> 1
    Char. 104: 0 --> 1
    Char. 106: 0 --> 1
    Char. 111: 1 --> 0
    Char. 115: 1 --> 0
    Char. 116: 1 --> 0
    Char. 117: 1 --> 0

Node 63 :
  All trees:
    Char. 30: 1 --> 2
    Char. 38: 1 --> 0
    Char. 95: 0 --> 1
    Char. 99: 0 --> 1

Node 64 :
  All trees:
    Char. 46: 0 --> 1
    Char. 51: 0 --> 1
    Char. 79: 0 --> 1

Node 65 :
  All trees:
    Char. 7: 0 --> 2
    Char. 12: 3 --> 2
    Char. 66: 1 --> 0
    Char. 136: 0 --> 1

Node 66 :
  All trees:
    Char. 11: 1 --> 2
    Char. 18: 0 --> 1
    Char. 28: 0 --> 1
    Char. 118: 0 --> 1
    Char. 138: 0 --> 1

Node 67 :
  All trees:
    Char. 18: 1 --> 2
    Char. 35: 0 --> 1
    Char. 55: 1 --> 0

Node 68 :
  All trees:
    Char. 2: 0 --> 1
    Char. 3: 0 --> 1
    Char. 10: 0 --> 1
    Char. 22: 0 --> 2

Node 69 :
  All trees:
    Char. 15: 0 --> 1
    Char. 31: 0 --> 1
    Char. 43: 2 --> 0
```

```
    Char. 55: 1 --> 0
    Char. 104: 1 --> 0
    Char. 105: 2 --> 1

Node 70 :
  All trees:
    Char. 2: 0 --> 2
    Char. 15: 0 --> 2
    Char. 55: 1 --> 0
    Char. 92: 1 --> 2
    Char. 120: 0 --> 1

Node 71 :
  All trees:
    Char. 5: 0 --> 2
    Char. 11: 2 --> 0
    Char. 18: 1 --> 3
    Char. 24: 0 --> 1
    Char. 34: 0 --> 2
    Char. 35: 0 --> 2
    Char. 87: 12 --> 3
    Char. 95: 0 --> 3
    Char. 96: 0 --> 1
    Char. 121: 0 --> 2
    Char. 122: 0 --> 1
    Char. 123: 0 --> 1
    Char. 124: 0 --> 1
    Char. 125: 0 --> 1

Node 72 :
  All trees:
    Char. 12: 1 --> 2
    Char. 24: 0 --> 1
    Char. 37: 1 --> 0
    Char. 77: 0 --> 1

Node 73 :
  All trees:
    Char. 9: 0 --> 1
    Char. 91: 0 --> 1
    Char. 104: 1 --> 0
    Char. 105: 2 --> 1
    Char. 127: 0 --> 1
    Char. 128: 0 --> 1
    Char. 133: 0 --> 1
    Char. 136: 0 --> 1
    Char. 141: 0 --> 1

Node 74 :
  All trees:
    Char. 11: 1 --> 0
    Char. 12: 3 --> 1
    Char. 98: 0 --> 1
    Char. 130: 0 --> 1

Node 75 :
  All trees:
    Char. 65: 0 --> 1
    Char. 101: 1 --> 2
    Char. 139: 0 --> 1

Node 76 :
  All trees:
    Char. 21: 1 --> 0
    Char. 32: 0 --> 1
    Char. 136: 0 --> 1

Node 77 :
  All trees:
    Char. 14: 1 --> 0
    Char. 103: 1 --> 0
    Char. 131: 0 --> 2

Node 78 :
  All trees:
    Char. 67: 0 --> 1
    Char. 71: 0 --> 1
    Char. 92: 1 --> 2
    Char. 118: 0 --> 1
  Some trees:
    Char. 65: 0 --> 2

Node 79 :
  All trees:
    Char. 73: 0 --> 1
    Char. 83: 0 --> 1
    Char. 91: 0 --> 1

Node 80 :
  All trees:
    Char. 62: 1 --> 0
    Char. 70: 1 --> 0
    Char. 82: 0 --> 2
    Char. 97: 2 --> 1
    Char. 103: 1 --> 0
    Char. 135: 0 --> 1
    Char. 157: 0 --> 1

Node 81 :
  Some trees:
    Char. 18: 0 --> 1
    Char. 65: 2 --> 1
```

```
      Char. 104: 1 --> 0
```

```
      Char. 158: 0 --> 1
```

Analysis 2

```
Synapomorphies common to 32 trees
(Node numbers refer to nodes in
consensus)

   Seymouriidae :
     All trees:
       No autapomorphies:

   Synapsida :
     All trees:
       Char. 11: 0 --> 1
       Char. 16: 0 --> 1
       Char. 102: 0 --> 1
       Char. 130: 0 --> 1
       Char. 186: 0 --> 1
       Char. 187: 0 --> 1

   Parareptilia :
     All trees:
       Char. 21: 0 --> 2
       Char. 34: 0 --> 1
       Char. 72: 0 --> 1
       Char. 129: 0 --> 1
       Char. 168: 0 --> 1
       Char. 170: 0 --> 1

   Captorhinidae :
     All trees:
       Char. 14: 0 --> 1
       Char. 33: 0 --> 1
       Char. 82: 0 --> 1
       Char. 91: 0 --> 1
       Char. 105: 0 --> 2

   Araeoscelidia :
     All trees:
       Char. 102: 0 --> 1
       Char. 105: 0 --> 1
       Char. 109: 0 --> 1
       Char. 139: 0 --> 1
       Char. 173: 0 --> 1
       Char. 186: 0 --> 1
       Char. 187: 0 --> 1
     Some trees:
       Char. 107: 0 --> 1

   Rhynchocephalia :
     All trees:
       Char. 64: 1 --> 0
       Char. 131: 0 --> 1
       Char. 142: 0 --> 1

   Squamata :
     All trees:
       Char. 18: 0 --> 1
       Char. 108: 0 --> 1
       Char. 109: 0 --> 1
       Char. 139: 0 --> 1
       Char. 144: 0 --> 1
       Char. 149: 0 --> 1
       Char. 150: 1 --> 2
     Some trees:
       Char. 17: 0 --> 2

   Prolacerta :
     All trees:
       Char. 17: 1 --> 0
       Char. 89: 1 --> 0
       Char. 125: 1 --> 0

   Trilophosaurus :
     All trees:
       Char. 6: 0 --> 1
       Char. 16: 2 --> 0
       Char. 21: 0 --> 2
       Char. 27: 1 --> 0
       Char. 40: 0 --> 12
       Char. 64: 1 --> 0
       Char. 81: 0 --> 1
       Char. 84: 1 --> 0
       Char. 98: 0 --> 1
       Char. 105: 2 --> 1
       Char. 113: 0 --> 1
       Char. 139: 0 --> 1
       Char. 142: 1 --> 0
       Char. 146: 1 --> 0
       Char. 163: 0 --> 1

   Rhynchosauria :
     All trees:
       Char. 15: 0 --> 1
       Char. 22: 1 --> 0
       Char. 83: 0 --> 1
       Char. 105: 2 --> 3
```

```
    Char. 109: 0 --> 1
    Char. 124: 0 --> 1
    Char. 129: 0 --> 1
    Char. 159: 1 --> 0
    Char. 165: 0 --> 1
    Char. 166: 0 --> 1
    Char. 173: 0 --> 1
    Char. 178: 0 --> 1

Archosauriformes :
  All trees:
    Char. 16: 2 --> 1
    Char. 19: 1 --> 0
    Char. 45: 0 --> 1
    Char. 80: 0 --> 1
    Char. 89: 1 --> 0
    Char. 95: 0 --> 1
    Char. 103: 1 --> 2
    Char. 118: 0 --> 1
    Char. 134: 0 --> 1
    Char. 138: 0 --> 1
    Char. 172: 1 --> 0

Claudiosaurus :
  All trees:
    Char. 20: 0 --> 1
    Char. 60: 0 --> 1
    Char. 61: 0 --> 1
    Char. 62: 0 --> 1
    Char. 63: 0 --> 1
    Char. 65: 2 --> 0
    Char. 90: 1 --> 2
    Char. 127: 0 --> 1
    Char. 134: 0 --> 1
    Char. 139: 0 --> 1
    Char. 142: 0 --> 1
    Char. 148: 1 --> 0
    Char. 173: 0 --> 1
    Char. 180: 0 --> 1

Coelurosauravus :
  All trees:
    Char. 17: 0 --> 1
    Char. 64: 1 --> 0
    Char. 72: 1 --> 0
    Char. 76: 1 --> 0
    Char. 78: 1 --> 0
    Char. 103: 1 --> 0
    Char. 108: 0 --> 1

Kuehneosauridae :
  All trees:
    No autapomorphies:

Acerosodontosaurus :
  All trees:
    Char. 131: 1 --> 0

Tangasaurus :
  Some trees:
    Char. 185: 0 --> 1

Youngina :
  All trees:
    Char. 66: 0 --> 1
    Char. 195: 0 --> 1

Thadeosaurus :
  All trees:
    Char. 63: 0 --> 2

Lanthanolania :
  All trees:
    Char. 126: 1 --> 0

Orovenator :
  All trees:
    Char. 12: 0 --> 1
    Char. 85: 0 --> 1
    Char. 127: 0 --> 1
    Char. 176: 0 --> 1

Sophineta :
  All trees:
    Char. 6: 0 --> 1
    Char. 39: 1 --> 0
    Char. 49: 1 --> 0
    Char. 173: 0 --> 1
    Char. 177: 0 --> 2
  Some trees:
    Char. 88: 1 --> 0
    Char. 130: 0 --> 1

Pamelina :
  All trees:
    Char. 95: 0 --> 1
    Char. 106: 1 --> 0
    Char. 154: 0 --> 1

Tanystropheus :
  All trees:
    Char. 9: 1 --> 0
```

```
      Char. 10: 0 --> 2
      Char. 17: 1 --> 2
      Char. 65: 2 --> 1
      Char. 75: 0 --> 1
      Char. 88: 0 --> 1
      Char. 98: 0 --> 1
      Char. 103: 1 --> 2
      Char. 134: 0 --> 1

Choristodera :
  All trees:
      Char. 1: 0 --> 1
      Char. 12: 0 --> 1
      Char. 16: 2 --> 1
      Char. 19: 1 --> 0
      Char. 20: 0 --> 1
      Char. 21: 0 --> 1
      Char. 32: 0 --> 1
      Char. 40: 0 --> 1
      Char. 44: 0 --> 1
      Char. 48: 0 --> 1
      Char. 49: 1 --> 0
      Char. 66: 0 --> 1
      Char. 74: 0 --> 1
      Char. 79: 0 --> 1
      Char. 81: 0 --> 1
      Char. 83: 0 --> 1
      Char. 91: 0 --> 1
      Char. 111: 0 --> 1
      Char. 113: 0 --> 1
      Char. 152: 0 --> 1
      Char. 175: 2 --> 1
      Char. 205: 0 --> 1
  Some trees:
      Char. 11: 0 --> 2
      Char. 168: 0 --> 1

Macrocnemus :
  All trees:
      Char. 12: 0 --> 1
      Char. 26: 1 --> 0
      Char. 28: 0 --> 1
      Char. 89: 1 --> 0
      Char. 106: 1 --> 0
      Char. 107: 0 --> 1
      Char. 147: 1 --> 0

Hovasaurus :
  All trees:
      Char. 63: 0 --> 2
      Char. 105: 0 --> 2

Pachypleurosaurs :
  All trees:
      Char. 11: 1 --> 0
      Char. 23: 0 --> 1
      Char. 33: 0 --> 1
      Char. 64: 1 --> 0
      Char. 101: 1 --> 0
      Char. 110: 0 --> 1
      Char. 201: 0 --> 1

Simosaurus :
  All trees:
      Char. 7: 1 --> 2
      Char. 9: 1 --> 0
      Char. 27: 1 --> 0
      Char. 120: 0 --> 1
      Char. 138: 1 --> 0

Placodus :
  All trees:
      Char. 2: 0 --> 1
      Char. 6: 0 --> 1
      Char. 35: 0 --> 1
      Char. 36: 1 --> 0
      Char. 39: 1 --> 0
      Char. 113: 1 --> 0
      Char. 115: 0 --> 1
      Char. 119: 1 --> 0
      Char. 121: 0 --> 1
      Char. 123: 0 --> 1
      Char. 151: 0 --> 1
  Some trees:
      Char. 3: 0 --> 1
      Char. 8: 3 --> 2
      Char. 19: 1 --> 0
      Char. 32: 0 --> 1
      Char. 46: 0 --> 1
      Char. 90: 2 --> 0
      Char. 91: 0 --> 1
      Char. 94: 2 --> 1
      Char. 116: 0 --> 1
      Char. 124: 0 --> 1
      Char. 138: 1 --> 2
      Char. 141: 1 --> 0
      Char. 152: 0 --> 1
      Char. 157: 1 --> 0
      Char. 164: 0 --> 1
      Char. 176: 0 --> 1
      Char. 190: 0 --> 1

Pistosauridae :
```

```
  All trees:
    Char. 18: 0 --> 1
    Char. 24: 0 --> 1
    Char. 38: 1 --> 0
    Char. 85: 0 --> 1
    Char. 90: 2 --> 1
    Char. 112: 0 --> 1
    Char. 177: 0 --> 1
    Char. 189: 0 --> 1
    Char. 199: 0 --> 1
  Some trees:
    Char. 60: 0 --> 1
    Char. 187: 2 --> 0

Askeptosaurus :
  All trees:
    Char. 2: 0 --> 1
    Char. 9: 1 --> 0
    Char. 34: 1 --> 0
    Char. 51: 1 --> 0
    Char. 65: 2 --> 1
    Char. 79: 0 --> 1
    Char. 165: 0 --> 1
    Char. 189: 0 --> 1

Clarazia :
  All trees:
    Char. 7: 1 --> 0
    Char. 8: 3 --> 0
    Char. 162: 2 --> 0
  Some trees:
    Char. 199: 1 --> 0

Thalattosaurus :
  All trees:
    Char. 42: 1 --> 0
    Char. 69: 1 --> 0
    Char. 99: 1 --> 0
    Char. 118: 0 --> 1
  Some trees:
    Char. 84: 0 --> 1

Helveticosaurus :
  All trees:
    Char. 38: 1 --> 0
    Char. 57: 1 --> 0
    Char. 62: 1 --> 0
    Char. 67: 1 --> 0
    Char. 78: 0 --> 1
    Char. 82: 0 --> 1
  Some trees:
    Char. 58: 1 --> 0
    Char. 69: 1 --> 0
    Char. 84: 1 --> 0
    Char. 105: 012 --> 3
    Char. 127: 0 --> 1
    Char. 190: 0 --> 1
    Char. 192: 1 --> 0
    Char. 195: 1 --> 0

Largocephalosaurus :
  All trees:
    Char. 12: 0 --> 1
    Char. 36: 1 --> 0
    Char. 194: 0 --> 23
  Some trees:
    Char. 45: 0 --> 1
    Char. 70: 1 --> 0
    Char. 130: 0 --> 1

Sinosaurosphargis :
  Some trees:
    Char. 8: 3 --> 4
    Char. 45: 0 --> 2
    Char. 130: 01 --> 0
    Char. 147: 1 --> 0
    Char. 186: 1 --> 0
    Char. 192: 1 --> 2
    Char. 193: 0 --> 2
    Char. 206: 0 --> 1

Wumengosaurus :
  All trees:
    Char. 51: 1 --> 0
    Char. 52: 0 --> 1
    Char. 64: 1 --> 0
    Char. 112: 0 --> 1
  Some trees:
    Char. 2: 0 --> 1
    Char. 48: 0 --> 1
    Char. 53: 0 --> 1
    Char. 77: 0 --> 1
    Char. 105: 012 --> 3
    Char. 118: 0 --> 2
    Char. 153: 1 --> 0
    Char. 191: 0 --> 1
    Char. 200: 0 --> 1
    Char. 203: 0 --> 1

Nanchangosaurus :
  All trees:
    No autapomorphies:
```

```
Hupehsuchus :
  All trees:
    Char. 6: 0 --> 1
    Char. 156: 1 --> 0

Chaohusaurus :
  All trees:
    Char. 6: 0 --> 1
    Char. 11: 1 --> 0
    Char. 51: 1 --> 0
    Char. 55: 0 --> 2
    Char. 75: 0 --> 1
    Char. 121: 0 --> 1
    Char. 183: 1 --> 0
    Char. 186: 1 --> 0

Utatsusaurus :
  All trees:
    Char. 8: 3 --> 1
    Char. 16: 2 --> 0
    Char. 21: 0 --> 1
    Char. 66: 1 --> 0
    Char. 84: 0 --> 1
    Char. 162: 2 --> 1
    Char. 166: 0 --> 1
    Char. 176: 0 --> 1

Eusaurosphargis :
  All trees:
    Char. 42: 1 --> 0
    Char. 54: 1 --> 0
    Char. 64: 1 --> 0
    Char. 66: 1 --> 0
    Char. 101: 1 --> 0
    Char. 112: 0 --> 1
    Char. 169: 0 --> 1
    Char. 172: 0 --> 1
    Char. 194: 0 --> 12
    Char. 198: 0 --> 1
  Some trees:
    Char. 85: 0 --> 1
    Char. 148: 1 --> 0
    Char. 175: 1 --> 2
    Char. 185: 0 --> 1
    Char. 190: 01 --> 0

Pomolispondylus_biani :
  Some trees:
    Char. 43: 0 --> 1
    Char. 59: 0 --> 1
    Char. 60: 2 --> 1
    Char. 69: 1 --> 0
    Char. 103: 2 --> 1
    Char. 118: 02 --> 1
    Char. 185: 0 --> 1
    Char. 188: 0 --> 1
    Char. 190: 01 --> 1
    Char. 206: 0 --> 1

Node 44 :
  All trees:
    No synapomorphies

Node 45 :
  All trees:
    Char. 30: 0 --> 1
    Char. 122: 1 --> 0
    Char. 126: 0 --> 1
    Char. 175: 0 --> 2
    Char. 182: 0 --> 1

Node 46 :
  All trees:
    Char. 110: 0 --> 1
    Char. 155: 0 --> 1
    Char. 183: 0 --> 1

Node 47 :
  All trees:
    Char. 7: 0 --> 1
    Char. 8: 0 --> 3
    Char. 9: 0 --> 1
    Char. 36: 0 --> 1
    Char. 54: 0 --> 1
    Char. 80: 0 --> 1
    Char. 96: 1 --> 0
    Char. 172: 0 --> 1

Node 48 :
  All trees:
    Char. 35: 0 --> 1
    Char. 171: 0 --> 1
  Some trees:
    Char. 71: 1 --> 0
    Char. 79: 0 --> 1
    Char. 80: 0 --> 1
    Char. 162: 1 --> 2
    Char. 169: 0 --> 1
    Char. 170: 0 --> 1
    Char. 175: 2 --> 1

Node 49 :
```

```
  All trees:
    Char. 0: 1 --> 0
    Char. 28: 0 --> 1
    Char. 126: 1 --> 0
  Some trees:
    Char. 63: 0 --> 2
    Char. 157: 1 --> 0
    Char. 176: 1 --> 0

Node 50 :
  All trees:
    Char. 65: 2 --> 0
    Char. 90: 1 --> 2
    Char. 127: 0 --> 1
    Char. 145: 1 --> 0
    Char. 146: 1 --> 0
  Some trees:
    Char. 37: 0 --> 3
    Char. 130: 1 --> 0

Node 51 :
  All trees:
    Char. 9: 1 --> 0
    Char. 24: 0 --> 1
    Char. 107: 0 --> 1
    Char. 140: 0 --> 1
    Char. 178: 0 --> 1
  Some trees:
    Char. 128: 0 --> 1

Node 52 :
  All trees:
    Char. 0: 0 --> 1
    Char. 25: 0 --> 1
    Char. 41: 0 --> 1
    Char. 64: 0 --> 1
    Char. 78: 0 --> 1
    Char. 131: 1 --> 0
    Char. 157: 0 --> 1
    Char. 176: 0 --> 1
  Some trees:
    Char. 76: 0 --> 1

Node 53 :
  All trees:
    Char. 147: 0 --> 1
    Char. 186: 0 --> 1
  Some trees:
    Char. 13: 0 --> 1
    Char. 14: 0 --> 1
    Char. 19: 0 --> 1
    Char. 70: 0 --> 1
    Char. 110: 1 --> 0
    Char. 185: 0 --> 1

Node 54 :
  All trees:
    Char. 184: 1 --> 0

Node 55 :
  All trees:
    Char. 5: 0 --> 1

Node 56 :
  All trees:
    Char. 16: 0 --> 2
    Char. 38: 0 --> 1
    Char. 90: 0 --> 1

Node 57 :
  All trees:
    Char. 0: 1 --> 0
    Char. 41: 1 --> 0
    Char. 74: 0 --> 1
    Char. 94: 2 --> 1
    Char. 102: 0 --> 1

Node 58 :
  All trees:
    Char. 1: 0 --> 1
    Char. 71: 1 --> 0
    Char. 79: 0 --> 1
    Char. 110: 0 --> 1
    Char. 112: 0 --> 1
    Char. 142: 0 --> 1
    Char. 170: 0 --> 2
    Char. 174: 0 --> 1
  Some trees:
    Char. 168: 0 --> 1

Node 59 :
  All trees:
    Char. 17: 0 --> 1
    Char. 54: 1 --> 0
    Char. 85: 0 --> 1
    Char. 181: 0 --> 1

Node 60 :
  All trees:
    Char. 8: 3 --> 1
    Char. 9: 1 --> 0
    Char. 35: 0 --> 1
```

```
      Char. 54: 0 --> 1
      Char. 65: 2 --> 0
      Char. 86: 0 --> 1
      Char. 140: 0 --> 1
      Char. 158: 0 --> 1
      Char. 177: 0 --> 1

Node 61 :
  All trees:
      Char. 87: 0 --> 1
      Char. 91: 0 --> 1
      Char. 92: 0 --> 1
      Char. 106: 1 --> 0
      Char. 153: 1 --> 0
      Char. 169: 0 --> 1
      Char. 175: 2 --> 1

Node 62 :
  All trees:
      Char. 1: 0 --> 1
      Char. 9: 0 --> 1
      Char. 45: 0 --> 1
      Char. 46: 0 --> 1
      Char. 83: 0 --> 1
      Char. 89: 1 --> 0
      Char. 103: 1 --> 2
      Char. 114: 0 --> 3
      Char. 178: 1 --> 0
      Char. 180: 0 --> 1
  Some trees:
      Char. 11: 0 --> 2
      Char. 17: 0 --> 2
      Char. 20: 0 --> 1
      Char. 37: 3 --> 0
      Char. 84: 0 --> 1

Node 63 :
  All trees:
      Char. 147: 0 --> 1
  Some trees:
      Char. 18: 0 --> 1
      Char. 90: 1 --> 0

Node 64 :
  All trees:
      Char. 104: 0 --> 1

Node 65 :
  All trees:
      Char. 67: 0 --> 1
      Char. 95: 0 --> 1
      Char. 178: 0 --> 1
      Char. 195: 0 --> 1

Node 66 :
  All trees:
      Char. 44: 0 --> 1
      Char. 48: 0 --> 1
      Char. 165: 0 --> 1
      Char. 166: 0 --> 1
  Some trees:
      Char. 43: 0 --> 1
      Char. 45: 01 --> 0
      Char. 118: 02 --> 2
      Char. 130: 01 --> 1
      Char. 148: 1 --> 0
      Char. 191: 02 --> 1

Node 67 :
  All trees:
      Char. 57: 0 --> 1
      Char. 66: 0 --> 1
      Char. 67: 0 --> 1
      Char. 73: 0 --> 1
      Char. 86: 0 --> 1
      Char. 90: 1 --> 2
      Char. 119: 0 --> 1
      Char. 144: 0 --> 1
  Some trees:
      Char. 11: 0 --> 1
      Char. 25: 1 --> 0
      Char. 70: 1 --> 12
      Char. 78: 1 --> 0
      Char. 103: 1 --> 2
      Char. 176: 1 --> 0
      Char. 204: 1 --> 0

Node 68 :
  All trees:
      Char. 49: 1 --> 0
      Char. 61: 0 --> 1
      Char. 62: 0 --> 1
      Char. 98: 0 --> 1
      Char. 113: 0 --> 1
      Char. 139: 0 --> 1
      Char. 150: 1 --> 2
      Char. 179: 0 --> 1
      Char. 185: 1 --> 0
  Some trees:
      Char. 129: 0 --> 1
      Char. 199: 0 --> 1
```

```
Node 69 :
  All trees:
    Char. 21: 0 --> 1
    Char. 40: 0 --> 1
    Char. 117: 0 --> 1
    Char. 127: 0 --> 1
  Some trees:
    Char. 8: 3 --> 2
    Char. 98: 0 --> 1
    Char. 116: 0 --> 1
    Char. 184: 1 --> 0

Node 70 :
  All trees:
    Char. 4: 0 --> 1
    Char. 18: 0 --> 1
    Char. 24: 0 --> 1
    Char. 28: 0 --> 1
    Char. 35: 0 --> 1
    Char. 93: 0 --> 1
    Char. 201: 0 --> 1

Node 71 :
  All trees:
    Char. 17: 1 --> 2
    Char. 32: 0 --> 1
    Char. 82: 0 --> 1
    Char. 88: 0 --> 1
    Char. 91: 0 --> 1
    Char. 115: 0 --> 1
    Char. 120: 0 --> 1
    Char. 121: 0 --> 1
    Char. 123: 0 --> 1
    Char. 183: 1 --> 0

Node 72 :
  All trees:
    Char. 196: 0 --> 1
  Some trees:
    Char. 103: 1 --> 2
    Char. 107: 0 --> 1
    Char. 151: 0 --> 2
    Char. 188: 0 --> 1
    Char. 190: 01 --> 2
    Char. 192: 01 --> 1
    Char. 201: 0 --> 1
    Char. 206: 0 --> 1
    Char. 210: 0 --> 1

Node 73 :
  All trees:
    Char. 34: 1 --> 0
    Char. 45: 01 --> 2
    Char. 153: 1 --> 2
    Char. 177: 0 --> 1
    Char. 207: 0 --> 1
    Char. 211: 0 --> 1
    Char. 212: 0 --> 1
  Some trees:
    Char. 2: 0 --> 1
    Char. 78: 0 --> 1
    Char. 103: 12 --> 1
    Char. 200: 0 --> 1
    Char. 203: 0 --> 1
    Char. 204: 0 --> 1
```

Analysis 3

```
Synapomorphies common to 6 trees
(Node numbers refer to nodes in
consensus)

Seymouriidae :
  All trees:
    No autapomorphies:

Synapsida :
  All trees:
    Char. 11: 0 --> 1
    Char. 16: 0 --> 1
    Char. 102: 0 --> 1
    Char. 130: 0 --> 1
    Char. 186: 0 --> 1
    Char. 187: 0 --> 1

Parareptilia :
  All trees:
    Char. 21: 0 --> 2
    Char. 34: 0 --> 1
    Char. 72: 0 --> 1
    Char. 129: 0 --> 1
    Char. 168: 0 --> 1
    Char. 170: 0 --> 1

Captorhinidae :
  All trees:
    Char. 14: 0 --> 1
    Char. 33: 0 --> 1
```

```
    Char. 82: 0 --> 1
    Char. 91: 0 --> 1
    Char. 105: 0 --> 2

Araeoscelidia :
  All trees:
    Char. 102: 0 --> 1
    Char. 105: 0 --> 1
    Char. 109: 0 --> 1
    Char. 139: 0 --> 1
    Char. 173: 0 --> 1
    Char. 186: 0 --> 1
    Char. 187: 0 --> 1
  Some trees:
    Char. 107: 0 --> 1

Rhynchocephalia :
  All trees:
    Char. 131: 0 --> 1
    Char. 142: 0 --> 1
  Some trees:
    Char. 64: 1 --> 0

Squamata :
  All trees:
    Char. 18: 0 --> 1
    Char. 108: 0 --> 1
    Char. 109: 0 --> 1
    Char. 139: 0 --> 1
    Char. 141: 1 --> 0
    Char. 144: 0 --> 1
    Char. 149: 0 --> 1
    Char. 150: 1 --> 2
  Some trees:
    Char. 17: 0 --> 2

Prolacerta :
  All trees:
    Char. 11: 2 --> 0
    Char. 100: 0 --> 1
    Char. 125: 1 --> 0
    Char. 178: 1 --> 0
  Some trees:
    Char. 107: 0 --> 2

Trilophosaurus :
  All trees:
    Char. 6: 0 --> 1
    Char. 16: 2 --> 0
    Char. 21: 0 --> 2
    Char. 27: 1 --> 0
    Char. 40: 0 --> 12
    Char. 64: 1 --> 0
    Char. 81: 0 --> 1
    Char. 84: 1 --> 0
    Char. 98: 0 --> 1
    Char. 99: 0 --> 1
    Char. 113: 0 --> 1
    Char. 139: 0 --> 1
    Char. 146: 1 --> 0
  Some trees:
    Char. 142: 1 --> 0
    Char. 163: 0 --> 1

Rhynchosauria :
  All trees:
    Char. 15: 0 --> 1
    Char. 22: 1 --> 0
    Char. 83: 0 --> 1
    Char. 109: 0 --> 1
    Char. 124: 0 --> 1
    Char. 159: 1 --> 0
    Char. 165: 0 --> 1
    Char. 166: 0 --> 1
    Char. 173: 0 --> 1
  Some trees:
    Char. 129: 0 --> 1

Archosauriformes :
  All trees:
    Char. 16: 2 --> 1
    Char. 19: 1 --> 0
    Char. 45: 0 --> 1
    Char. 80: 0 --> 1
    Char. 89: 1 --> 0
    Char. 95: 0 --> 1
    Char. 103: 1 --> 2
    Char. 118: 0 --> 1
    Char. 134: 0 --> 1
    Char. 138: 0 --> 1
    Char. 172: 1 --> 0
    Char. 178: 1 --> 0

Claudiosaurus :
  All trees:
    Char. 47: 0 --> 1
    Char. 60: 0 --> 1
    Char. 61: 0 --> 1
    Char. 62: 0 --> 1
    Char. 63: 0 --> 1
    Char. 84: 0 --> 1
    Char. 134: 0 --> 1
```

```
    Char. 142: 0 --> 1
    Char. 173: 0 --> 1
    Char. 180: 0 --> 1
  Some trees:
    Char. 20: 0 --> 1
    Char. 65: 2 --> 0
    Char. 127: 0 --> 1
    Char. 139: 0 --> 1
    Char. 148: 1 --> 0

Coelurosauravus :
  All trees:
    Char. 0: 0 --> 1
    Char. 17: 0 --> 1
    Char. 72: 1 --> 0
    Char. 103: 1 --> 0
    Char. 108: 0 --> 1
  Some trees:
    Char. 3: 0 --> 1
    Char. 64: 1 --> 0
    Char. 76: 1 --> 0
    Char. 176: 0 --> 1

Kuehneosauridae :
  All trees:
    No autapomorphies:

Acerosodontosaurus :
  All trees:
    Char. 131: 1 --> 0

Tangasaurus :
  Some trees:
    Char. 185: 0 --> 1

Youngina :
  All trees:
    Char. 66: 0 --> 1
    Char. 195: 0 --> 1

Thadeosaurus :
  All trees:
    Char. 63: 0 --> 2

Lanthanolania :
  All trees:
    Char. 126: 1 --> 0

Orovenator :
  All trees:
    Char. 12: 0 --> 1
    Char. 85: 0 --> 1
    Char. 127: 0 --> 1
    Char. 176: 0 --> 1

Sophineta :
  All trees:
    Char. 6: 0 --> 1
    Char. 39: 1 --> 0
    Char. 49: 1 --> 0
    Char. 88: 1 --> 0
    Char. 173: 0 --> 1
    Char. 177: 0 --> 2
  Some trees:
    Char. 94: 12 --> 0
    Char. 130: 0 --> 1

Pamelina :
  All trees:
    Char. 95: 0 --> 1
    Char. 106: 1 --> 0
    Char. 154: 0 --> 1

Tanystropheus :
  All trees:
    Char. 10: 0 --> 2
    Char. 11: 2 --> 1
    Char. 65: 2 --> 1
    Char. 75: 0 --> 1
    Char. 88: 0 --> 1
    Char. 98: 0 --> 1
    Char. 103: 1 --> 2
    Char. 134: 0 --> 1
    Char. 172: 1 --> 0
  Some trees:
    Char. 162: 1 --> 2

Choristodera :
  All trees:
    Char. 0: 0 --> 1
    Char. 7: 1 --> 2
    Char. 12: 0 --> 1
    Char. 16: 2 --> 1
    Char. 19: 1 --> 0
    Char. 21: 0 --> 1
    Char. 24: 0 --> 1
    Char. 32: 0 --> 1
    Char. 40: 0 --> 1
    Char. 42: 1 --> 0
    Char. 44: 0 --> 1
    Char. 48: 0 --> 1
    Char. 49: 1 --> 0
```

```
    Char. 66: 0 --> 1
    Char. 81: 0 --> 1
    Char. 83: 0 --> 1
    Char. 91: 0 --> 1
    Char. 101: 1 --> 0
    Char. 113: 0 --> 1
    Char. 125: 1 --> 0
    Char. 135: 1 --> 0
    Char. 141: 1 --> 0
    Char. 152: 0 --> 1
    Char. 205: 0 --> 1
  Some trees:
    Char. 107: 0 --> 1
    Char. 163: 0 --> 1
    Char. 175: 2 --> 1

Macrocnemus :
  All trees:
    Char. 12: 0 --> 1
    Char. 26: 1 --> 0
    Char. 28: 0 --> 1
    Char. 106: 1 --> 0
    Char. 147: 1 --> 0
  Some trees:
    Char. 107: 0 --> 1

Hovasaurus :
  All trees:
    Char. 63: 0 --> 2
    Char. 105: 0 --> 2

Pachypleurosaurs :
  All trees:
    Char. 11: 1 --> 0
    Char. 23: 0 --> 1
    Char. 33: 0 --> 1
    Char. 64: 1 --> 0
    Char. 101: 1 --> 0
    Char. 110: 0 --> 1
    Char. 201: 0 --> 1

Simosaurus :
  All trees:
    Char. 7: 1 --> 2
    Char. 9: 1 --> 0
    Char. 27: 1 --> 0
    Char. 120: 0 --> 1
    Char. 138: 1 --> 0

Placodus :
  All trees:
    Char. 2: 0 --> 1
    Char. 6: 0 --> 1
    Char. 19: 1 --> 0
    Char. 32: 0 --> 1
    Char. 35: 0 --> 1
    Char. 36: 1 --> 0
    Char. 39: 1 --> 0
    Char. 90: 2 --> 0
    Char. 91: 0 --> 1
    Char. 115: 0 --> 1
    Char. 121: 0 --> 1
    Char. 123: 0 --> 1
    Char. 124: 0 --> 1
    Char. 138: 1 --> 2
    Char. 141: 1 --> 0
    Char. 151: 0 --> 1
    Char. 152: 0 --> 1
    Char. 157: 1 --> 0
    Char. 164: 0 --> 1
    Char. 176: 0 --> 1
  Some trees:
    Char. 3: 0 --> 1
    Char. 7: 1 --> 0
    Char. 94: 2 --> 1
    Char. 154: 0 --> 1
    Char. 190: 0 --> 1
    Char. 192: 0 --> 1

Pistosauridae :
  All trees:
    Char. 18: 0 --> 1
    Char. 24: 0 --> 1
    Char. 38: 1 --> 0
    Char. 85: 0 --> 1
    Char. 90: 2 --> 1
    Char. 112: 0 --> 1
    Char. 177: 0 --> 1
    Char. 187: 2 --> 0
    Char. 189: 0 --> 1
    Char. 199: 0 --> 1

Askeptosaurus :
  All trees:
    Char. 2: 0 --> 1
    Char. 9: 1 --> 0
    Char. 47: 0 --> 1
    Char. 51: 1 --> 0
    Char. 79: 0 --> 1
    Char. 165: 0 --> 1
  Some trees:
    Char. 17: 2 --> 1
```

```
Clarazia :
  All trees:
    Char. 7: 1 --> 0
    Char. 21: 1 --> 0
    Char. 162: 2 --> 0
  Some trees:
    Char. 199: 1 --> 0

Thalattosaurus :
  All trees:
    Char. 42: 1 --> 0
    Char. 84: 0 --> 1
    Char. 99: 1 --> 0
    Char. 118: 0 --> 1
  Some trees:
    Char. 69: 1 --> 0

Helveticosaurus :
  All trees:
    Char. 38: 1 --> 0
    Char. 65: 2 --> 1
    Char. 69: 1 --> 0
    Char. 82: 0 --> 1
    Char. 127: 0 --> 1
  Some trees:
    Char. 7: 1 --> 0
    Char. 78: 0 --> 1
    Char. 105: 1 --> 3
    Char. 106: 1 --> 0
    Char. 168: 0 --> 1
    Char. 190: 0 --> 1

Largocephalosaurus :
  All trees:
    Char. 12: 0 --> 1
    Char. 36: 1 --> 0
    Char. 70: 1 --> 0
    Char. 194: 0 --> 23

Sinosaurosphargis :
  All trees:
    Char. 130: 1 --> 0
    Char. 147: 1 --> 0
    Char. 154: 0 --> 1
    Char. 186: 1 --> 0
    Char. 192: 1 --> 2
  Some trees:
    Char. 193: 0 --> 2

Wumengosaurus :
  All trees:
    Char. 51: 1 --> 0
    Char. 52: 0 --> 1
    Char. 53: 0 --> 1
    Char. 118: 0 --> 2
  Some trees:
    Char. 2: 0 --> 1
    Char. 20: 0 --> 1
    Char. 48: 0 --> 1
    Char. 64: 1 --> 0
    Char. 77: 0 --> 1
    Char. 103: 1 --> 2
    Char. 112: 0 --> 1
    Char. 184: 1 --> 0
    Char. 191: 0 --> 1
    Char. 200: 0 --> 1
    Char. 203: 0 --> 1
    Char. 204: 1 --> 0

Nanchangosaurus :
  All trees:
    No autapomorphies:

Hupehsuchus :
  All trees:
    Char. 6: 0 --> 1
    Char. 156: 1 --> 0

Chaohusaurus :
  All trees:
    Char. 6: 0 --> 1
    Char. 51: 1 --> 0
    Char. 55: 0 --> 2
    Char. 75: 0 --> 1
    Char. 121: 0 --> 1
    Char. 183: 1 --> 0
    Char. 186: 1 --> 0
  Some trees:
    Char. 11: 1 --> 0
    Char. 101: 0 --> 1

Utatsusaurus :
  All trees:
    Char. 16: 2 --> 0
    Char. 21: 0 --> 1
    Char. 84: 0 --> 1
    Char. 162: 2 --> 1
    Char. 166: 0 --> 1
    Char. 176: 0 --> 1

Eusaurosphargis :
  All trees:
```

```
    Char. 42: 1 --> 0
    Char. 172: 0 --> 1
    Char. 194: 0 --> 12
    Char. 198: 0 --> 1
  Some trees:
    Char. 45: 0 --> 1
    Char. 47: 0 --> 1
    Char. 54: 1 --> 0
    Char. 64: 1 --> 0
    Char. 70: 1 --> 2
    Char. 84: 0 --> 1
    Char. 85: 0 --> 1
    Char. 101: 1 --> 0
    Char. 106: 1 --> 0
    Char. 112: 0 --> 1
    Char. 168: 0 --> 1
    Char. 169: 0 --> 1
    Char. 192: 0 --> 1
    Char. 195: 0 --> 1

Pomolispondylus_biani :
  All trees:
    Char. 190: 0 --> 1
    Char. 194: 0 --> 1

Node 44 :
  All trees:
    No synapomorphies

Node 45 :
  All trees:
    Char. 30: 0 --> 1
    Char. 122: 1 --> 0
    Char. 126: 0 --> 1
    Char. 175: 0 --> 2
    Char. 182: 0 --> 1

Node 46 :
  All trees:
    Char. 110: 0 --> 1
    Char. 155: 0 --> 1
    Char. 183: 0 --> 1

Node 47 :
  All trees:
    Char. 7: 0 --> 1
    Char. 8: 0 --> 3
    Char. 9: 0 --> 1
    Char. 36: 0 --> 1
    Char. 54: 0 --> 1
    Char. 80: 0 --> 1
    Char. 96: 1 --> 0
    Char. 172: 0 --> 1

Node 48 :
  All trees:
    Char. 35: 0 --> 1
    Char. 171: 0 --> 1
  Some trees:
    Char. 42: 1 --> 0
    Char. 71: 1 --> 0
    Char. 79: 0 --> 1
    Char. 80: 0 --> 1
    Char. 169: 0 --> 1
    Char. 170: 0 --> 1
    Char. 175: 2 --> 1

Node 49 :
  All trees:
    Char. 28: 0 --> 1
    Char. 126: 1 --> 0
  Some trees:
    Char. 63: 0 --> 2
    Char. 78: 0 --> 1
    Char. 101: 1 --> 0

Node 50 :
  All trees:
    Char. 7: 1 --> 0
    Char. 145: 1 --> 0
    Char. 146: 1 --> 0
  Some trees:
    Char. 37: 0 --> 3
    Char. 65: 2 --> 0
    Char. 107: 0 --> 1
    Char. 127: 0 --> 1
    Char. 130: 1 --> 0

Node 51 :
  All trees:
    Char. 41: 0 --> 1
    Char. 42: 0 --> 1
    Char. 131: 1 --> 0
    Char. 135: 0 --> 1
  Some trees:
    Char. 25: 0 --> 1
    Char. 64: 0 --> 1
    Char. 76: 0 --> 1
    Char. 140: 0 --> 1
    Char. 157: 0 --> 1
    Char. 178: 0 --> 1
```

```
Node 52 :
  All trees:
    Char. 186: 0 --> 1
  Some trees:
    Char. 8: 3 --> 1
    Char. 13: 0 --> 1
    Char. 14: 0 --> 1
    Char. 19: 0 --> 1
    Char. 70: 0 --> 1
    Char. 90: 1 --> 2
    Char. 94: 0 --> 2
    Char. 105: 0 --> 1
    Char. 110: 1 --> 0
    Char. 147: 0 --> 1
    Char. 172: 1 --> 0
    Char. 185: 0 --> 1

Node 53 :
  All trees:
    Char. 184: 1 --> 0

Node 54 :
  All trees:
    Char. 5: 0 --> 1

Node 55 :
  All trees:
    Char. 16: 0 --> 2
    Char. 38: 0 --> 1
    Char. 90: 0 --> 1

Node 56 :
  All trees:
    Char. 8: 1 --> 3
    Char. 54: 1 --> 0
    Char. 140: 1 --> 0
    Char. 173: 0 --> 2
  Some trees:
    Char. 20: 1 --> 0
    Char. 130: 1 --> 0

Node 57 :
  All trees:
    Char. 71: 1 --> 0
    Char. 110: 0 --> 1
    Char. 138: 1 --> 0
    Char. 170: 0 --> 2
    Char. 174: 0 --> 1
    Char. 187: 0 --> 1

Node 58 :
  All trees:
    Char. 1: 0 --> 1
    Char. 74: 0 --> 1
    Char. 79: 0 --> 1
    Char. 90: 2 --> 1
    Char. 172: 0 --> 1
  Some trees:
    Char. 11: 0 --> 2
    Char. 47: 0 --> 1
    Char. 78: 0 --> 1
    Char. 84: 0 --> 1
    Char. 88: 1 --> 0
    Char. 168: 0 --> 1
    Char. 176: 0 --> 1

Node 59 :
  All trees:
    Char. 35: 0 --> 1
    Char. 65: 2 --> 0
    Char. 86: 0 --> 1
    Char. 158: 0 --> 1
    Char. 177: 0 --> 1
  Some trees:
    Char. 162: 1 --> 2

Node 60 :
  All trees:
    Char. 87: 0 --> 1
    Char. 91: 0 --> 1
    Char. 92: 0 --> 1
    Char. 100: 0 --> 1
    Char. 106: 1 --> 0
    Char. 169: 0 --> 1
  Some trees:
    Char. 107: 0 --> 2
    Char. 175: 2 --> 1

Node 61 :
  All trees:
    Char. 1: 0 --> 1
    Char. 11: 0 --> 2
    Char. 45: 0 --> 1
    Char. 46: 0 --> 1
    Char. 83: 0 --> 1
    Char. 84: 0 --> 1
    Char. 89: 1 --> 0
    Char. 103: 1 --> 2
    Char. 114: 0 --> 3
    Char. 178: 1 --> 0
    Char. 180: 0 --> 1
  Some trees:
```

```
    Char. 9: 0 --> 1
    Char. 17: 0 --> 2
    Char. 37: 3 --> 0
    Char. 129: 1 --> 0

Node 62 :
  All trees:
    Char. 147: 0 --> 1
  Some trees:
    Char. 18: 0 --> 1
    Char. 90: 1 --> 0

Node 63 :
  All trees:
    Char. 104: 0 --> 1

Node 64 :
  All trees:
    Char. 0: 0 --> 1
    Char. 67: 0 --> 1
    Char. 74: 1 --> 0
    Char. 95: 0 --> 1
    Char. 99: 0 --> 1
    Char. 195: 0 --> 1

Node 65 :
  All trees:
    Char. 43: 0 --> 1
    Char. 44: 0 --> 1
    Char. 48: 0 --> 1
    Char. 118: 0 --> 2
    Char. 126: 1 --> 0
    Char. 165: 0 --> 1
    Char. 166: 0 --> 1
    Char. 191: 2 --> 1
  Some trees:
    Char. 45: 1 --> 0
    Char. 148: 1 --> 0

Node 66 :
  All trees:
    Char. 37: 0 --> 1
    Char. 53: 0 --> 1
    Char. 59: 0 --> 1
    Char. 205: 0 --> 1
  Some trees:
    Char. 58: 0 --> 1
    Char. 84: 0 --> 1
    Char. 168: 01 --> 0
    Char. 187: 0 --> 2
    Char. 195: 0 --> 1

Node 67 :
  All trees:
    Char. 21: 0 --> 1
    Char. 25: 1 --> 0
    Char. 40: 0 --> 1
    Char. 98: 0 --> 1
    Char. 117: 0 --> 1
    Char. 127: 0 --> 1
    Char. 184: 1 --> 0

Node 68 :
  All trees:
    Char. 4: 0 --> 1
    Char. 18: 0 --> 1
    Char. 24: 0 --> 1
    Char. 28: 0 --> 1
    Char. 35: 0 --> 1
    Char. 78: 0 --> 1
    Char. 90: 2 --> 1
    Char. 201: 0 --> 1
  Some trees:
    Char. 25: 0 --> 1
    Char. 54: 1 --> 0
    Char. 93: 0 --> 1

Node 69 :
  All trees:
    Char. 21: 0 --> 1
    Char. 114: 0 --> 1
    Char. 143: 1 --> 0
  Some trees:
    Char. 17: 0 --> 2
    Char. 98: 0 --> 1
    Char. 153: 0 --> 1
    Char. 176: 0 --> 1

Node 70 :
  All trees:
    Char. 32: 0 --> 1
    Char. 82: 0 --> 1
    Char. 88: 0 --> 1
    Char. 91: 0 --> 1
    Char. 115: 0 --> 1
    Char. 120: 0 --> 1
    Char. 121: 0 --> 1
    Char. 123: 0 --> 1
    Char. 183: 1 --> 0

Node 71 :
  All trees:
    Char. 103: 1 --> 2
```

```
      Char. 188: 0 --> 1
      Char. 196: 0 --> 2

  Node 72 :
    All trees:
      Char. 43: 0 --> 1
      Char. 59: 0 --> 1
      Char. 192: 0 --> 1
    Some trees:
      Char. 69: 1 --> 0

  Node 73 :
    All trees:
      Char. 107: 0 --> 1
      Char. 151: 0 --> 2
      Char. 188: 0 --> 1
      Char. 190: 0 --> 2
      Char. 192: 0 --> 1
      Char. 196: 0 --> 1
      Char. 201: 0 --> 1
      Char. 206: 0 --> 1
      Char. 210: 0 --> 1
    Some trees:
      Char. 103: 1 --> 2

  Node 74 :
    All trees:
      Char. 3: 0 --> 1
      Char. 205: 0 --> 2
      Char. 208: 0 --> 1
      Char. 209: 0 --> 1
    Some trees:
      Char. 30: 1 --> 0
      Char. 33: 0 --> 1
      Char. 69: 1 --> 0
      Char. 98: 0 --> 1
      Char. 130: 1 --> 0
      Char. 184: 01 --> 1
      Char. 189: 0 --> 1
      Char. 202: 0 --> 1

  Node 75 :
    All trees:
      Char. 45: 0 --> 2
      Char. 78: 0 --> 1
      Char. 177: 0 --> 1
      Char. 207: 0 --> 1
      Char. 211: 0 --> 1
      Char. 212: 0 --> 1
    Some trees:
      Char. 2: 0 --> 1
      Char. 34: 1 --> 0
      Char. 103: 12 --> 1
      Char. 153: 1 --> 2
      Char. 200: 0 --> 1
      Char. 203: 0 --> 1
      Char. 204: 0 --> 1
```

References

Bickelmann C, Müller J, Reisz RR. 2009. The enigmatic diapsid Acerosodontosaurus piveteaui (Reptilia: Neodiapsida) from the Upper Permian of Madagascar and the paraphyly of "younginiform" reptiles. *Canadian Journal of Earth Sciences* **46**:651–661. DOI 10.1139/E09-038.

Chen X, Motani R, Cheng L, Jiang D, Rieppel OC. 2014. The enigmatic marine reptile *Nanchangosaurus* from the Lower Triassic of Hubei, China and the phylogenetic affinities of Hupehsuchia. *PLoS ONE* **9**:e102361. DOI 10.1371/journal.pone.0102361.

deBraga M, Rieppel OC. 1997. Reptile phylogeny and the interrelationships of turtles. *Zoological Journal of the Linnean Society* **120**:281–354. DOI 10.1006/zjls.1997.0079.

Dilkes DW. 1998. The Early Triassic rhynchosaur *Mesosuchus Browni* and the interrelationships of basal archosauromorph reptiles. *Philosophical Transactions of the Royal Society of London B: Biological Sciences* **353**:501–541. DOI 10.1098/rstb.1998.0225.

Edinger T. 1935. *Pistosaurus*. *Neues Jahrbuch für Geologie und Paläontologie, Abhandlungen* **74**:321–359.

Goloboff PA. 1999. Analyzing large data sets in reasonable times: Solutions for composite optima. *Cladistics* **15**:415–428. DOI 10.1006/clad.1999.0122.

Goloboff PA, Catalano SA. 2016. TNT version 1.5, including a full implementation of phylogenetic morphometrics. *Cladistics* **32**:221–238. DOI 10.1111/cla.12160.

Kass RE, Raftery AE. 1995. Bayes factors. *Journal of the American Statistical Association* **90**:773–795. DOI 10.2307/2291091.

Kuhn-Schnyder E. 1971. Über einen Schädel von *Askeptosaurus italicus* Nopcsa aus der Mittleren Trias des Monte San Giorgio (Kt. Tessin, Schweiz). *Abhandlungen des Hessischen Landesamtes für Bodenforschung* **60**:89–98.

Li C, Jiang D-Y, Cheng L, Wu X-C, Rieppel O. 2014. A new species of *largocephalosaurus* (diapsida: Saurosphargidae), with implications for the morphological diversity and phylogeny of the group. *Geological Magazine* **151**:100–120. DOI 10.1017/S001675681300023X.

Li C, Rieppel O, Wu X-C, Zhao L-J, Wang L-T. 2011. A new Triassic marine reptile from southwestern China. *Journal of Vertebrate Paleontology* **31**:303–312. DOI 10.1080/02724634.2011.550368.

Maddison WP, Maddison DR. 2021. Mesquite: A modular system for evolutionary analysis.

Müller J. 2004. The relationships among diapsid reptiles and the influence of taxon selection. In: Arratia G, Wilson MVH, Cloutier R eds. *Recent advances in the origin and early radiation of vertebrates*. Munich: Verlag Dr. Friedrich Pfeil, 379–408.

Müller J, Scheyer TM, Head JJ, Barrett PM, Werneburg I, Ericson PGP, Pol D, Sánchez-Villagra MR. 2010. Homeotic effects, somitogenesis and the evolution of vertebral numbers in recent and fossil amniotes. *Proceedings of the National Academy of Sciences* **107**:2118–2123. DOI 10.1073/pnas.0912622107.

Nicholls EL. 1999. A reexamination of *Thalattosaurus* and *Nectosaurus* and the relationships of the Thalattosauria (Reptilia: Diapsida). *PaleoBios* **19**:1–29.

Nixon KC. 1999. The parsimony ratchet, a new method for rapid parsimony analysis. *Cladistics* **15**:407–414. DOI 10.1006/clad.1999.0121.

Plummer M, Best N, Cowles K, Vines K. 2006. CODA: Convergence diagnosis and output analysis for MCMC. *R News* **6**:7–11.

R Core Team. 2021. R: A language and environment for statistical computing. Vienna, Austria: R Foundation for Statistical Computing.

Rieppel OC, Mazin J-M, Tchernov E. 1999. Sauropterygia from the Middle Triassic of Makhtesh Ramon, Negev, Israel. *Fieldiana Geology* **40**:1–85.

Ronquist F, Teslenko M, Mark P van der, Ayres DL, Darling A, Höhna S, Larget B, Liu L, Suchard MA, Huelsenbeck JP. 2012. MrBayes 3.2: Efficient Bayesian phylogenetic inference and model choice across a large model space. *Systematic Biology* **61**:539–542. DOI 10.1093/sysbio/sys029.

Sato T, Cheng Y-N, Wu X-C, Li C. 2010. Osteology of *Yunguisaurus* Cheng et al., 2006 (Reptilia; Sauropterygia), a Triassic pistosauroid from China. *Paleontological Research* **14**:179–195. DOI 10.2517/1342-8144-14.3.179.

Scheyer TM, Neenan JM, Bodogan T, Furrer H, Obrist C, Plamondon M. 2017. A new, exceptionally preserved juvenile specimen of *Eusaurosphargis dalsassoi* (diapsida) and implications for mesozoic marine diapsid phylogeny. *Scientific Reports* **7**:1–22. DOI 10.1038/s41598-017-04514-x.

Storrs GW. 1997. Morphological and taxonomic clarification of the genus *Plesiosaurus*. In: Callaway JM, Nicholls EL eds. *Ancient Marine Reptiles*. San Diego, California: Academic Press, 501.

Storrs GW, Taylor MA. 1996. Cranial anatomy of a new plesiosaur genus from the lowermost Lias (Rhaetian/Hettangian) of Street, Somerset, England. *Journal of Vertebrate Paleontology* **16**:403–420. DOI 10.1080/02724634.1996.10011330.

Wu X-C, Cheng Y-N, Li C, Zhao L-J, Sato T. 2011. New information on *Wumengosaurus delicatomandibularis* Jiang et al., 2008 (Diapsida: Sauropterygia), with a revision of the osteology and phylogeny of the taxon. *Journal of Vertebrate Paleontology* **31**:70–83. DOI 10.1080/02724634.2011.546724.

Xie W, Lewis PO, Fan Y, Kuo L, Chen M-H. 2011. Improving marginal likelihood estimation for Bayesian phylogenetic model selection. *Systematic Biology* **60**:150–160. DOI 10.1093/sysbio/syq085.

Supplemental Figures

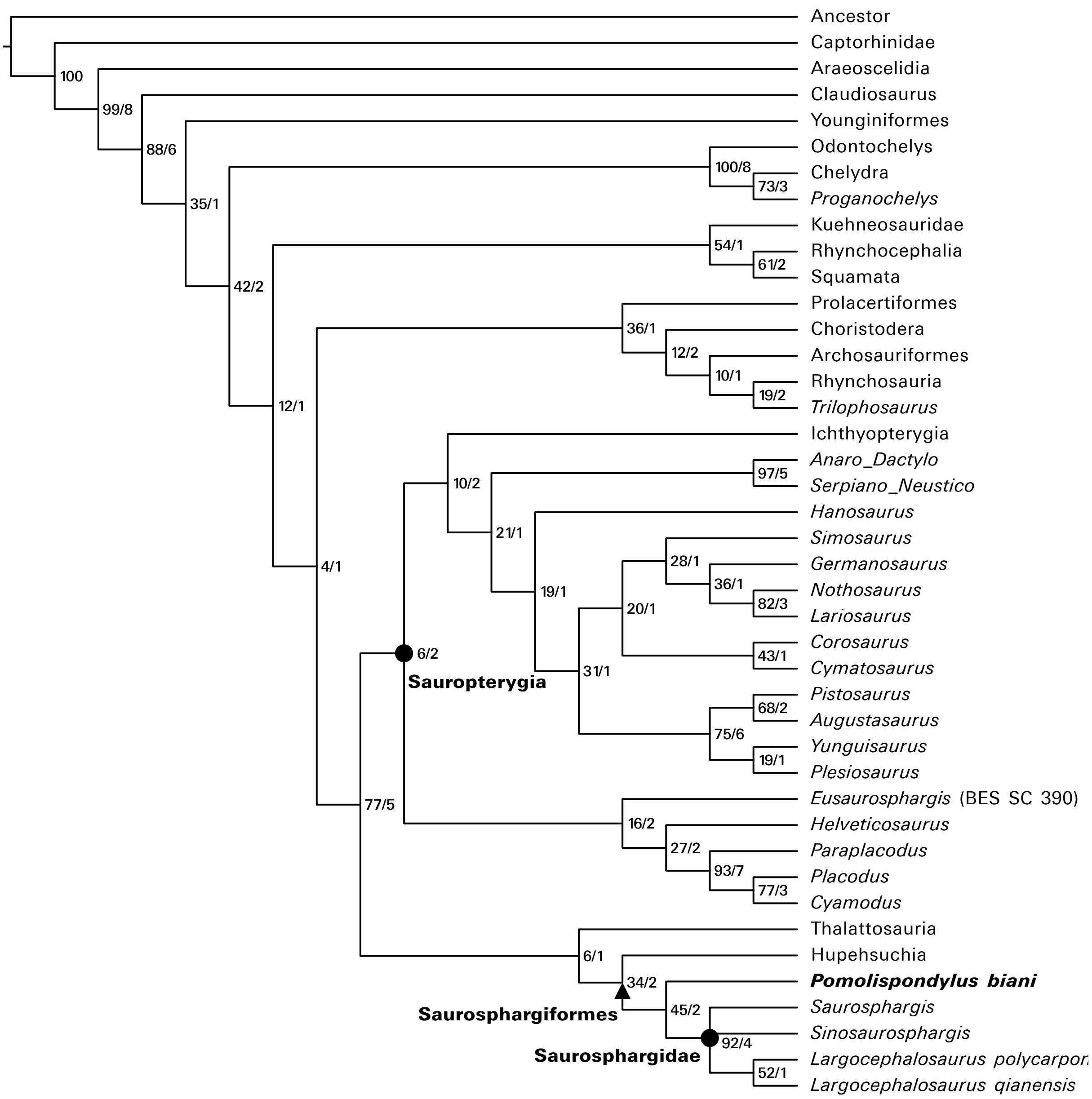

Figure S1 Analysis 1 in TNT of modified matrix from Li et al. (2014) with 42 OTUs. Strict consensus of 2 trees each with length 608 steps. Bootstrap (%)/Bremer support values are indicated at each node.

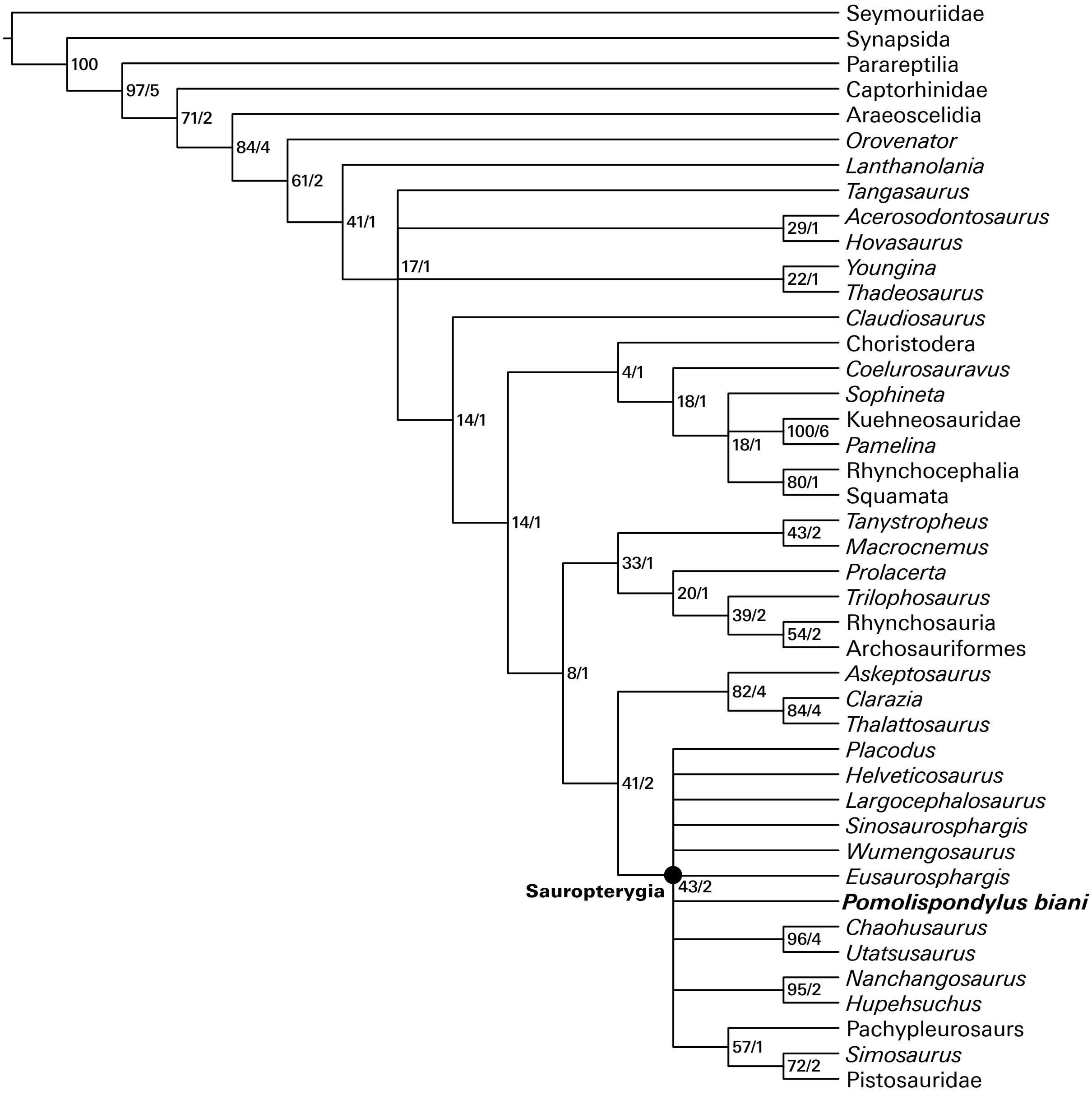

Figure S2 Analysis 2 in TNT of modified matrix from Scheyer et al. (2017) with 43 OTUs. Strict consensus of 32 trees each with length 851 steps. Bootstrap (%)/Bremer support values are indicated at each node.

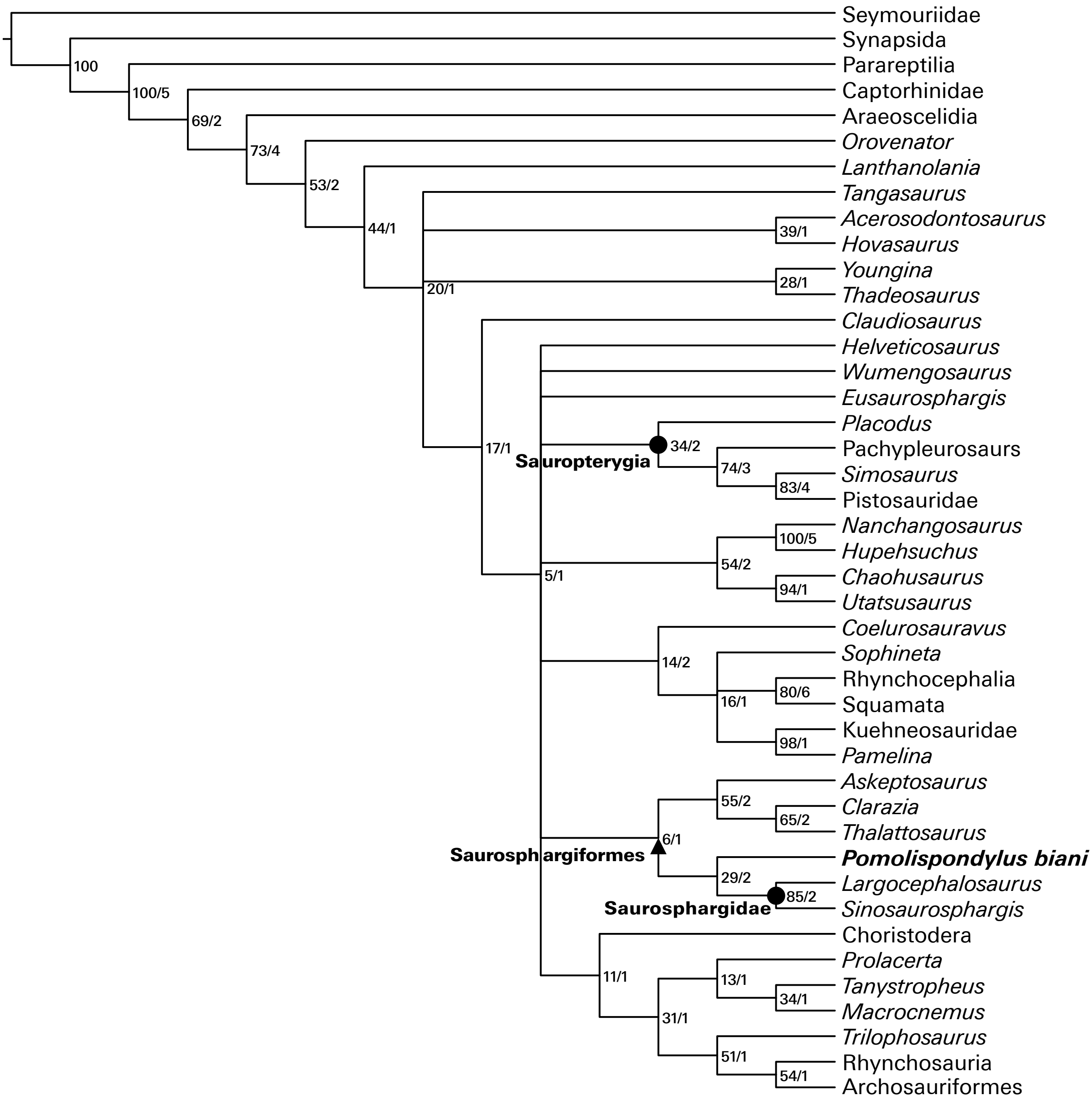

Figure S3 Analysis 3 in TNT of modified matrix from Scheyer et al. (2017) with 43 OTUs and marine characters coded as uncertain. Strict consensus of 6 trees each with length 814 steps. Bootstrap (%)/Bremer support values are indicated at each node.

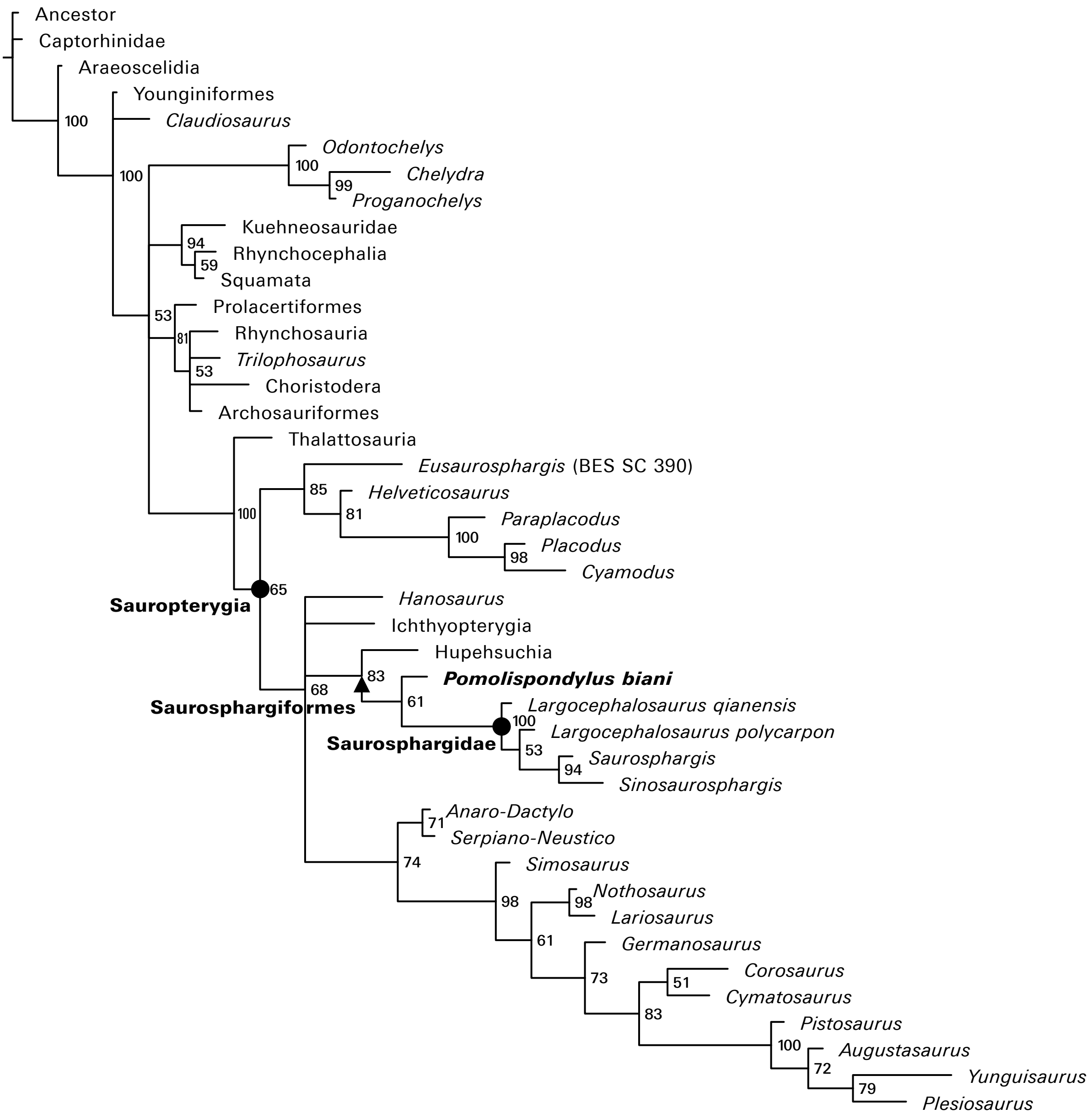

Figure S4 Analysis 1 in MrBayes with 42 operational taxonomic units from the modified matrix of Li et al. (2014) and gamma-distribution rates model 50% majority-rule consensus tree. Clade credibility values (%) are indicated at each node.

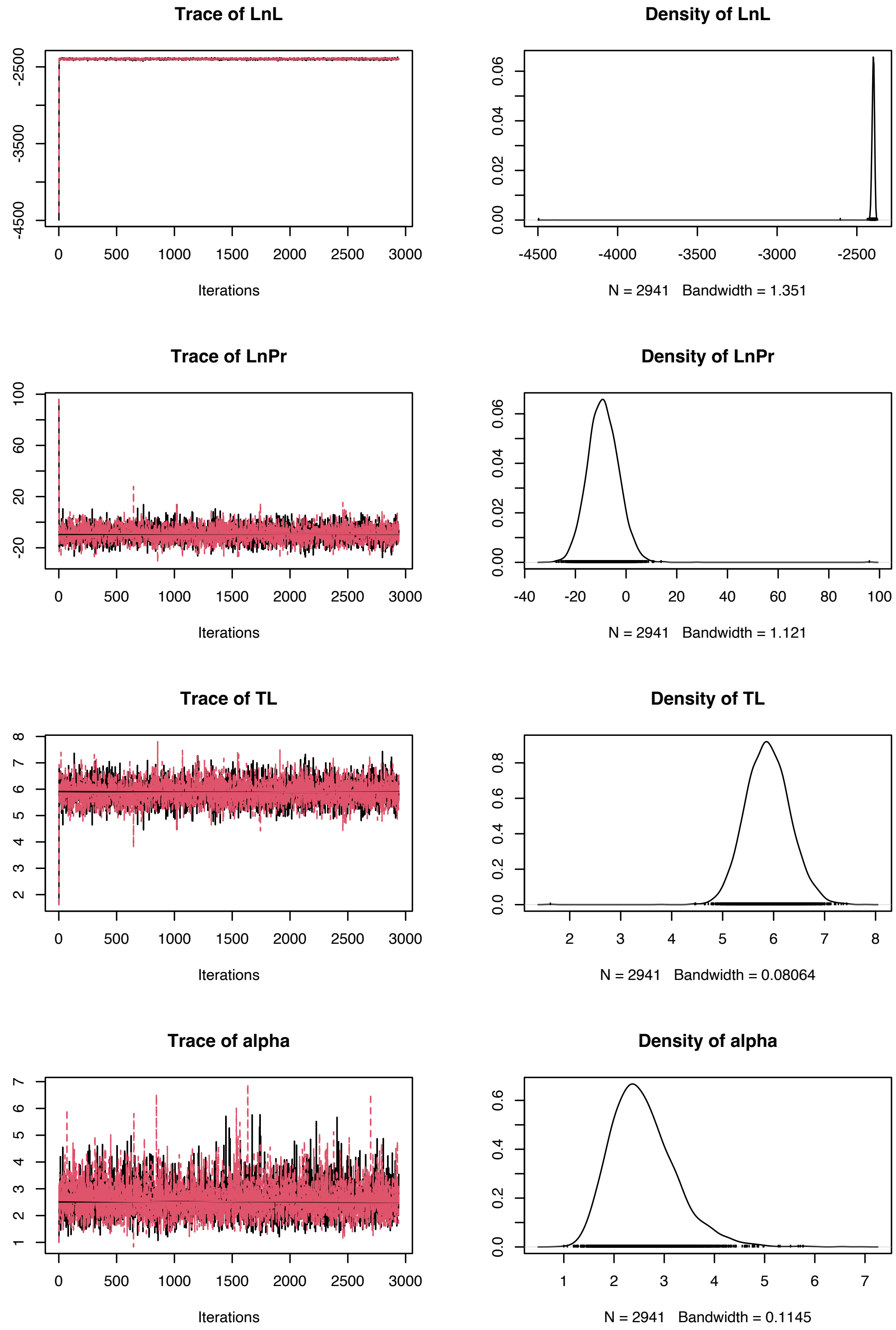

Figure S5 Traceplots and posterior distributions of parameters estimated in Analysis 1 in MrBayes with gamma-distribution rates model.

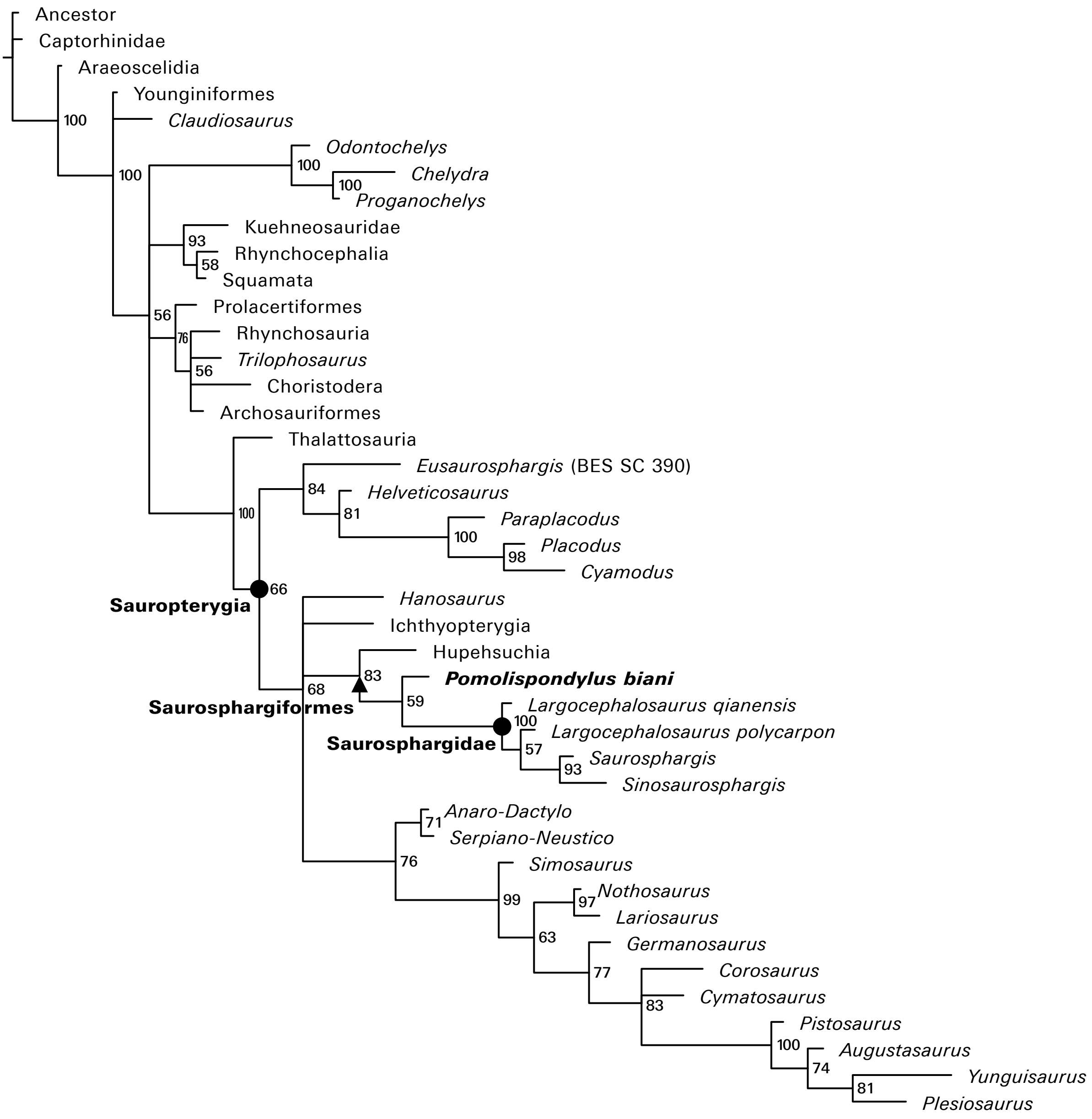

Figure S6 Analysis 1 in MrBayes with 42 operational taxonomic units from the modified matrix of Li et al. (2014) and log-normal-distribution rates model 50% majority-rule consensus tree. Clade credibility values (%) are indicated at each node.

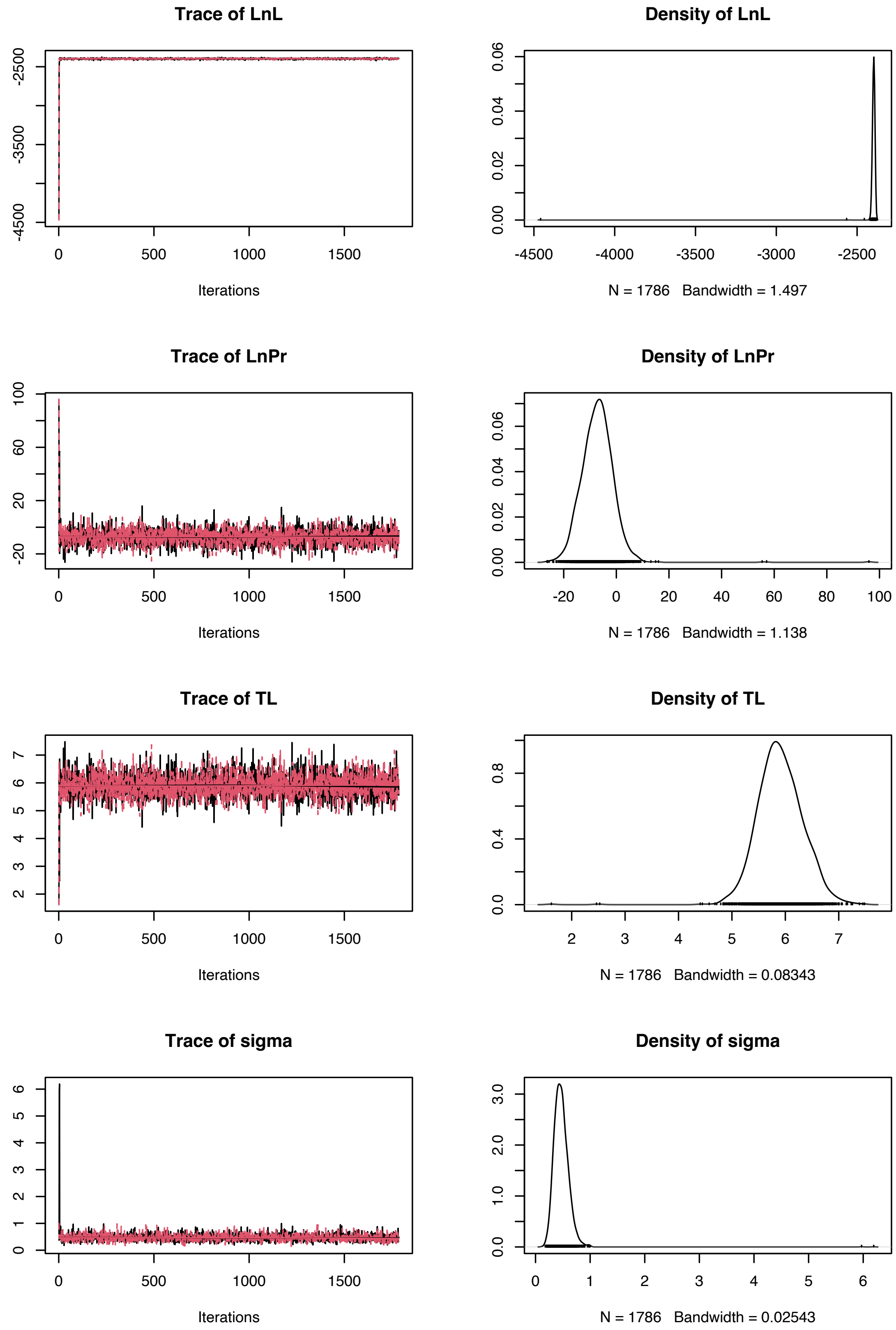

Figure S7 Traceplots and posterior distributions of parameters estimated in Analysis 1 in MrBayes with log-normal-distribution rates model.

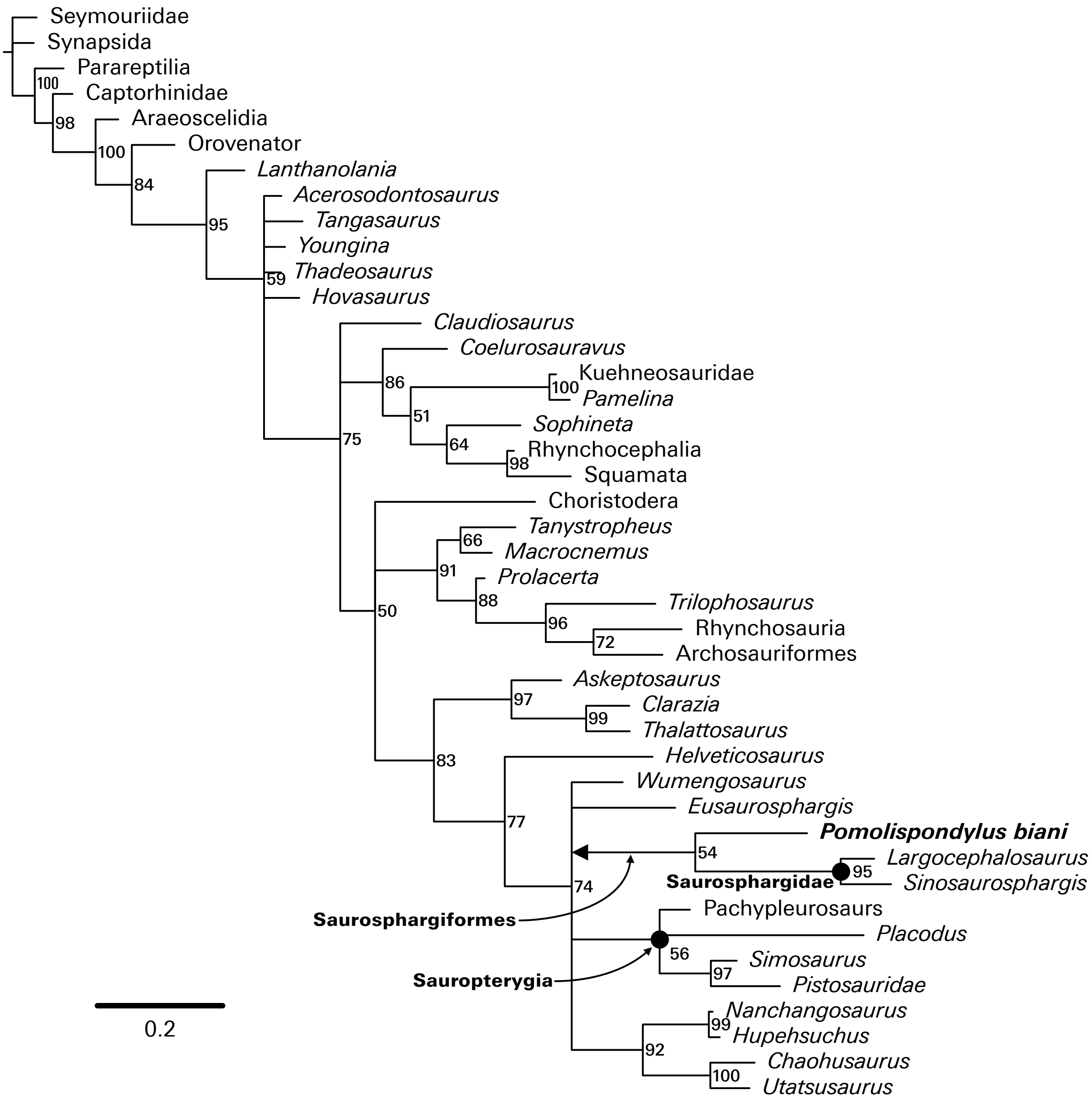

Figure S8 Analysis 2 in MrBayes with 43 operational taxonomic units from the modified matrix of Scheyer et al. (2017) and gamma-distribution rates model 50% majority-rule consensus tree. Clade credibility values (%) are indicated at each node.

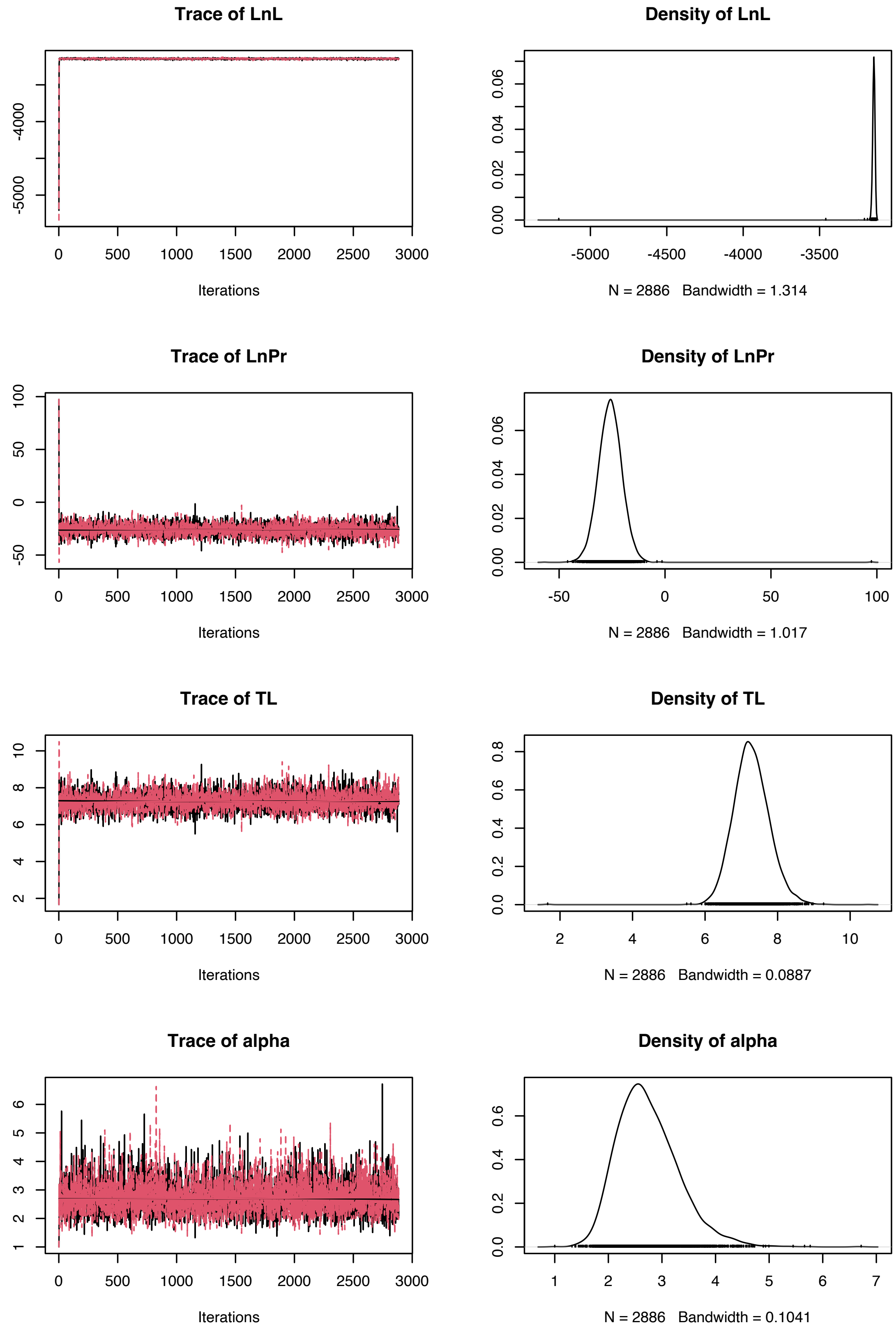

Figure S9 Traceplots and posterior distributions of parameters estimated in Analysis 2 in MrBayes with gamma-distribution rates model.

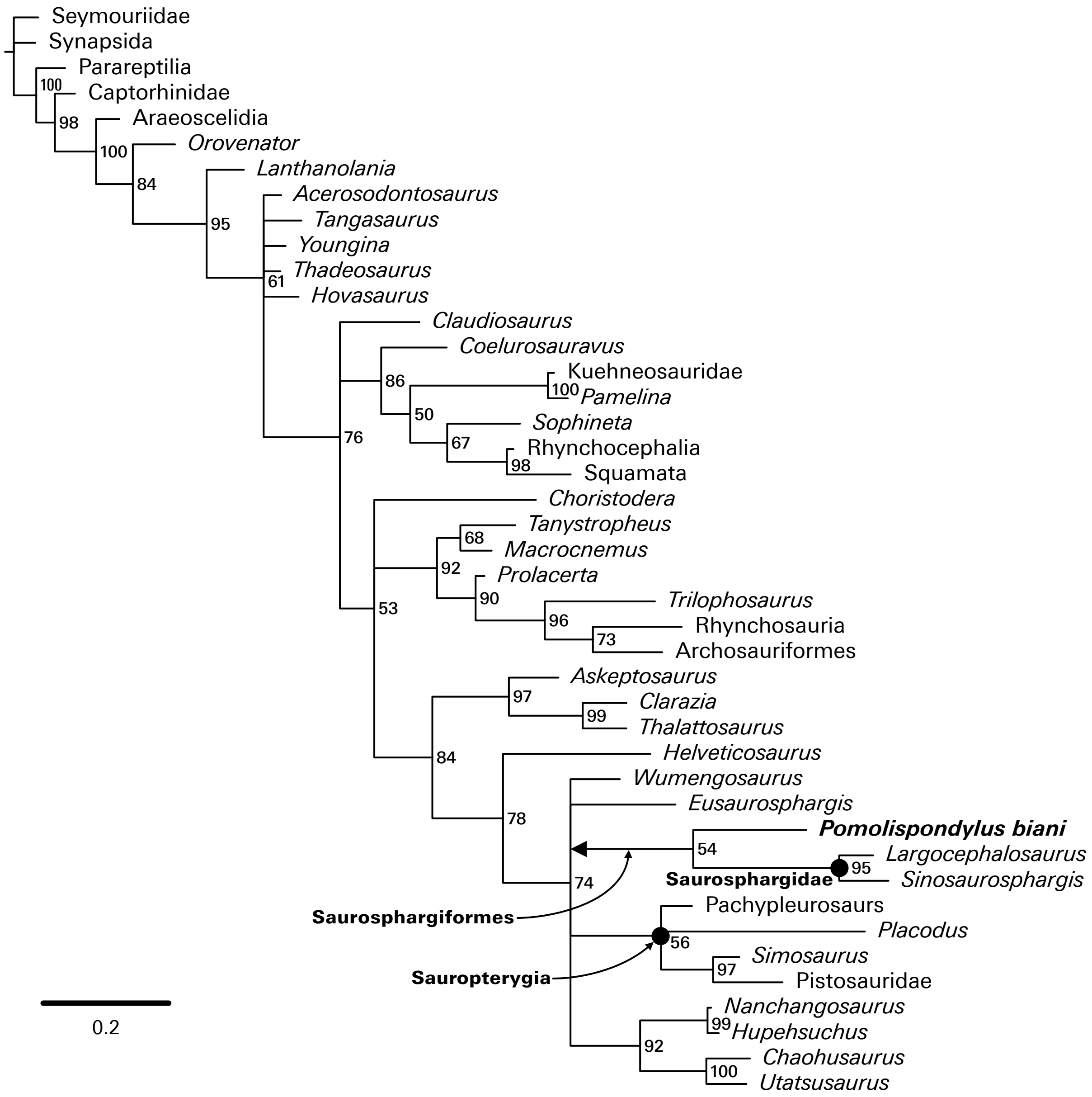

Figure S10 Analysis 2 in MrBayes with 53 operational taxonomic units from the modified matrix of Scheyer et al. (2017) and log-normal-distribution rates model 50% majority-rule consensus tree. Clade credibility values (%) are indicated at each node.

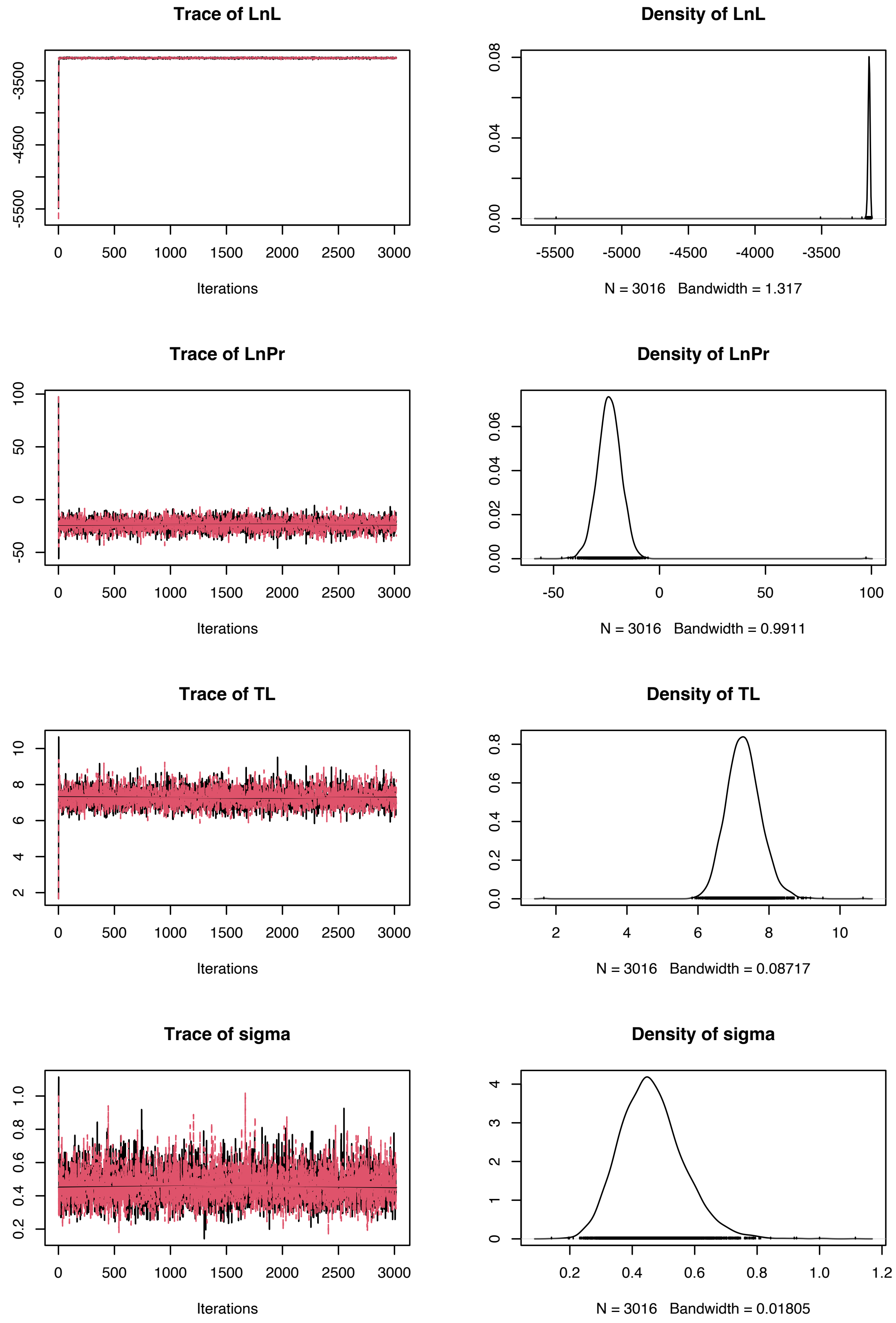

Figure S11 Traceplots and posterior distributions of parameters estimated in Analysis 2 in MrBayes with log-normal-distribution rates model.

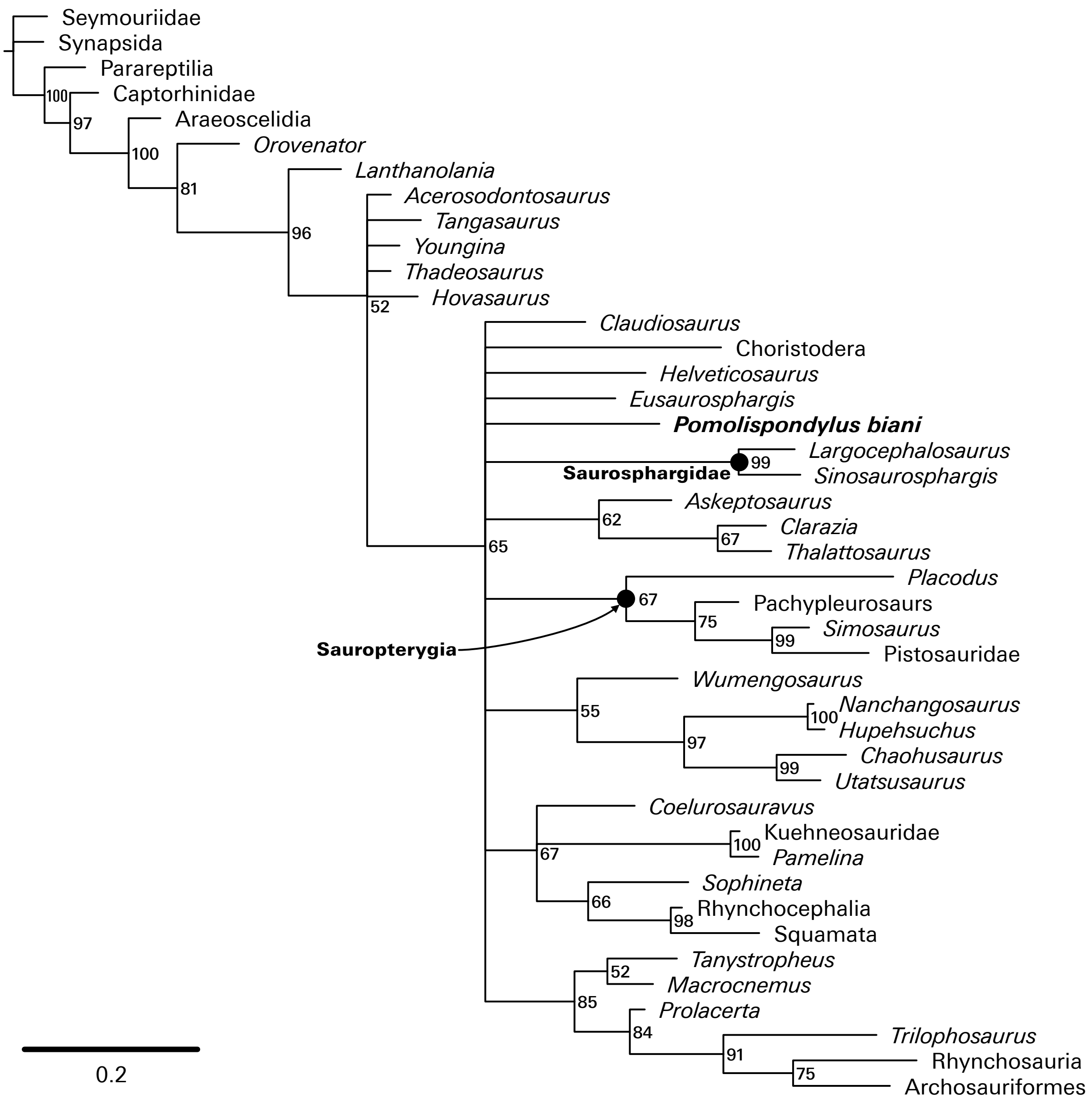

Figure S12 Analysis 3 in MrBayes with 43 operational taxonomic units from the modified matrix of Scheyer et al. (2017) with marine characters coded as uncertainty and gamma-distribution rates model 50% majority-rule consensus tree. Clade credibility values (%) are indicated at each node.

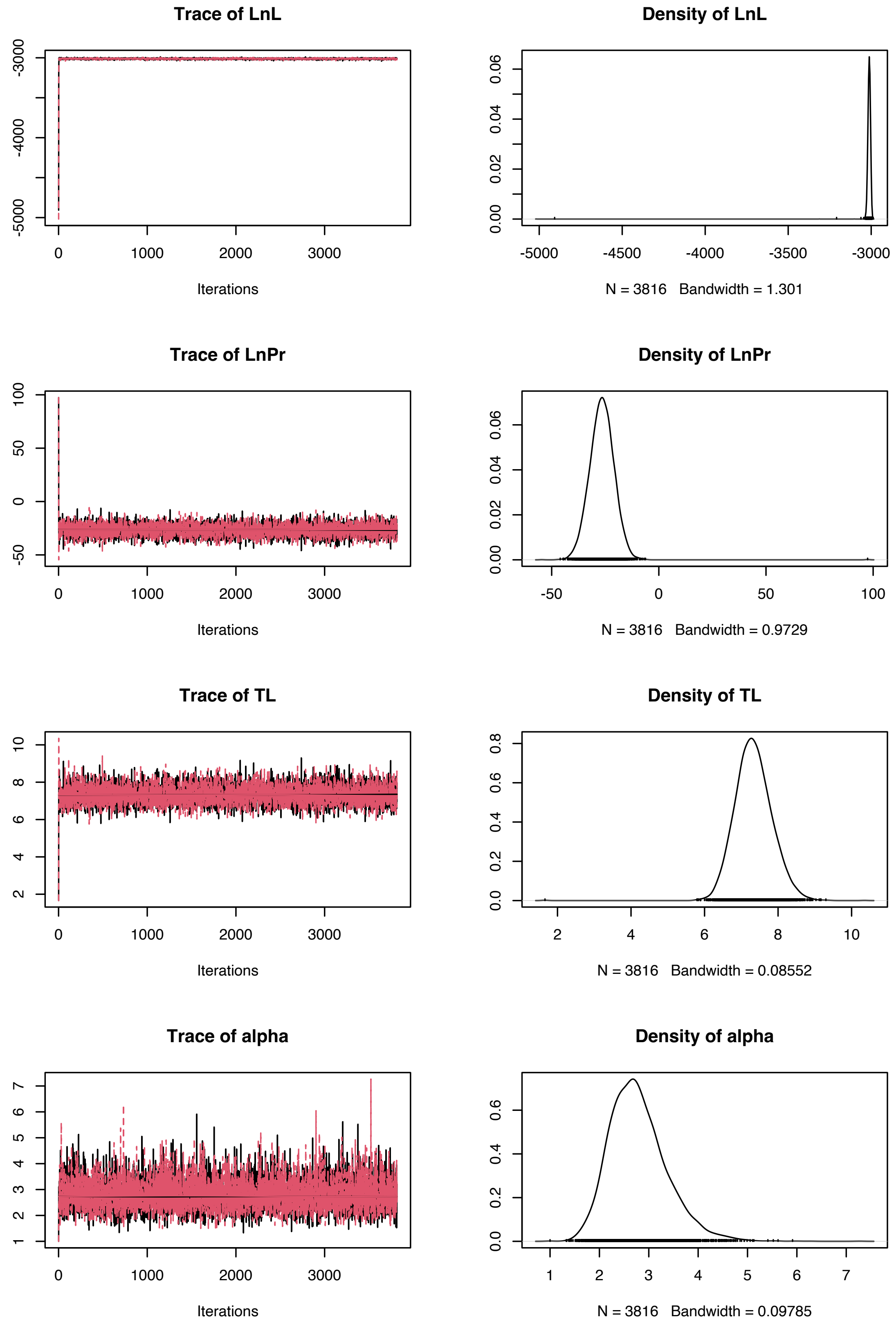

Figure S13 Traceplots and posterior distributions of parameters estimated in Analysis 3 in MrBayes with gamma-distribution rates model.

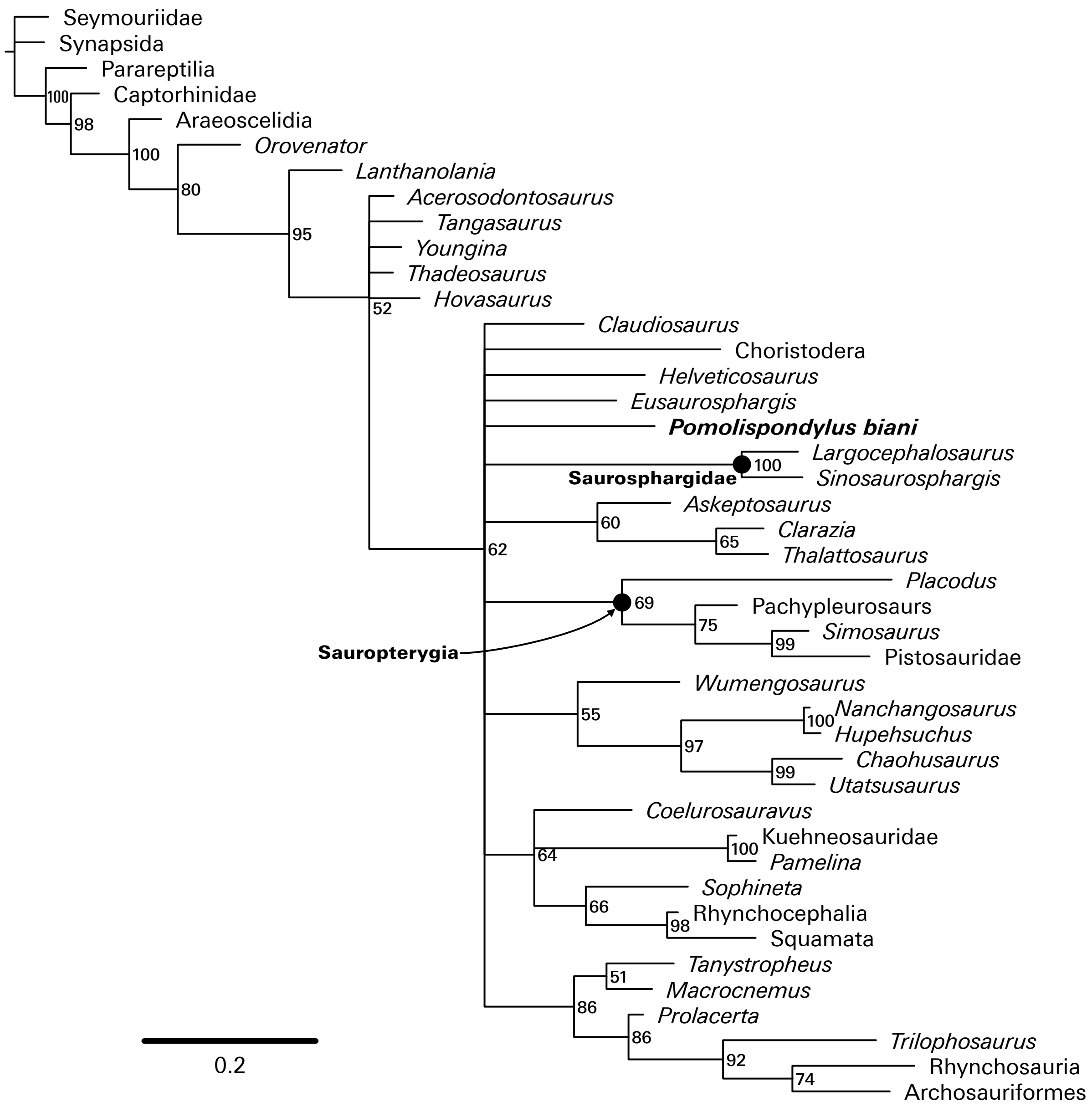

Figure S14 Analysis 3 in MrBayes with 43 operational taxonomic units from the modified matrix of Scheyer et al. (2017) with marine characters coded as uncertainty and log-normal-distribution rates model 50% majority-rule consensus tree. Clade credibility values (%) are indicated at each node.

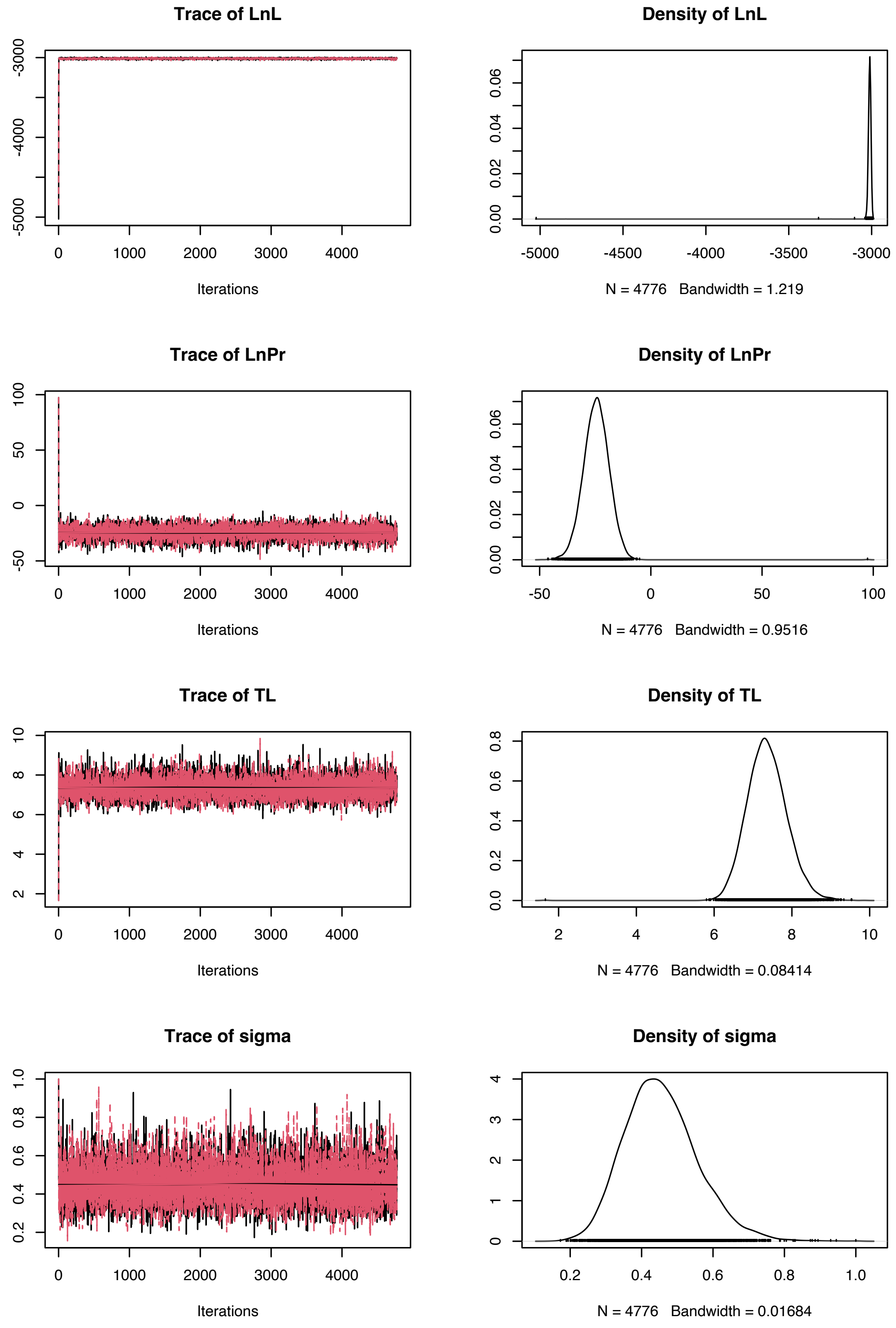

Figure S15 Traceplots and posterior distributions of parameters estimated in Analysis 3 in MrBayes with log-normal-distribution rates model.
